# *De novo* assembly and characterization of central nervous system transcriptome reveals neurotransmitter signaling systems in the rice striped stem borer, *Chilo suppressalis*

**DOI:** 10.1186/s12864-015-1742-7

**Published:** 2015-07-15

**Authors:** Gang Xu, Shun-Fan Wu, Ya-Su Wu, Gui-Xiang Gu, Qi Fang, Gong-Yin Ye

**Affiliations:** State Key Laboratory of Rice Biology & Key Laboratory of Agricultural Entomology of Ministry of Agriculture, Institute of Insect Sciences, Zhejiang University, Hangzhou, 310058 China; State & Local Joint Engineering Research Center of Green Pesticide Invention and Application, College of Plant Protection, Nanjing Agricultural University, Nanjing, 210095 China; Institute of Insect Sciences, Zhejiang University, Hangzhou, 310058 China

**Keywords:** Transcriptome, Central nervous system, Neurotransmitter signaling

## Abstract

**Background:**

Neurotransmitter signaling systems play crucial roles in multiple physiological and behavioral processes in insects. Genome wide analyses of *de novo* transcriptome sequencing and gene specific expression profiling provide rich resources for studying neurotransmitter signaling pathways. The rice striped stem borer, *Chilo suppressalis* is a destructive rice pest in China and other Asian countries. The characterization of genes involved in neurotransmitter biosynthesis and transport could identify potential targets for disruption of the neurochemical communication and for crop protection.

**Results:**

Here we report *de novo* sequencing of the *C. suppressalis* central nervous system transcriptome, identification and expression profiles of genes putatively involved in neurotransmitter biosynthesis, packaging, and recycling/degradation. A total of 54,411 unigenes were obtained from the transcriptome analysis. Among these unigenes, we have identified 32 unigenes (31 are full length genes), which encode 21 enzymes and 11 transporters putatively associated with biogenic aminergic signaling, acetylcholinergic signaling, glutamatergic signaling and GABAergic signaling. RT-PCR and qRT-PCR results indicated that 12 enzymes were highly expressed in the central nervous system and all the transporters were expressed at significantly high levels in the central nervous system. In addition, the transcript abundances of enzymes and transporters in the central nervous system were validated by qRT-PCR. The high expression levels of these genes suggest their important roles in the central nervous system.

**Conclusions:**

Our study identified genes potentially involved in neurotransmitter biosynthesis and transport in *C. suppressalis* and these genes could serve as targets to interfere with neurotransmitter production. This study presents an opportunity for the development of specific and environmentally safe insecticides for pest control.

**Electronic supplementary material:**

The online version of this article (doi:10.1186/s12864-015-1742-7) contains supplementary material, which is available to authorized users.

## Background

In both invertebrates and vertebrates, the control of physiology and behavior is achieved through the use of neurotransmitter signaling. While all tissues undoubtedly participate in this chemical communication, the central nervous system is a particularly rich source of neurotransmitter signaling compounds [1]. Neurotransmitters, as messengers in chemical synaptic transmission, play crucial roles in information transfer in the central nervous system [2]. In general, neurotransmitters consist of acetylcholine (ACh), glutamate (Glu), γ-aminobutyric acid (GABA), and monoamines such as dopamine (DA), octopamine (OA), tyramine (TA), serotonin (5-HT) and histamine (HA). Neurotransmitters are always produced via various synthetases in the presynaptic terminal to regulate multiple physiological and behavioral processes. Subsequently, neurotransmitters generated in the neurons are antiported by protons into synaptic vesicles using vesicular neurotransmitter transporters. Interactions of vesicles with the neurosynapse membrane occur when the calcium level inside the cell changes. These processes are assisted by vesicle-associated membrane proteins. The neurotransmitters are then released into synaptic cleft by fusion of the vesicles and cell membranes, which then interact with neurotransmitter receptors located in postsynaptic membranes [3]. In insects, the actions of neurotransmitters have been shown to be mainly mediated via the activation of ligand-gated ion channels and related G protein-coupled receptors (GPCRs) [4, 5]. A common feature of GPCR activation is the subsequent change of the intracellular concentration of second messengers, including cAMP and Ca^2+^. Some GPCRs bind to Gαs/Gαi proteins, which then interact with adenylyl cyclase (AC) in the plasma membrane to increase or decrease the concentration of cAMP. Some other GPCRs bind to Gαq subunits and activate phospholipase C (PLC) activity, causing a rise of [Ca^2+^]_i_ [6]. In brief, the effects of the neurotransmitters are mediated through interactions with corresponding G protein-coupled receptors (GPCRs) to alter the concentrations of second messengers in the signaling pathways, resulting in modulation of various physiological processes. The actions of neurotransmitters are terminated by their reuptake to the cytosol via neurotransmitter transporters.

The rice striped stem borer, *Chilo suppressalis* (Walker) (Lepidoptera: Crambidae) is one of the most economically important rice pests in Asia, northern Africa, and southern Europe. It causes serious crop loss every year, particularly in China because of rice cultivation and the popularization of hybrid varieties. To date, chemical control is still the major method to protect rice from damage by the rice stem borer. Unfortunately, *C. suppressalis* has developed resistance to many chemical insecticides and the estimated cost for controlling this pest is approximately 160,000,000 US dollars annually [7]. Compared to 10 years ago, the pesticides currently used in controlling the borers are much different. This is due to the evolution of multiple resistances to several classes of commonly used insecticides, including nereistoxin analogues, organochlorines, organophosphates, pyrethroids, and phenylpyrazoles [8]. The development of insecticide resistance in rice striped stem borer is the primary reason for insecticide replacement, along with the introduction of new chemical insecticides with higher activity [9]. Recently, some insecticides with novel mode of actions, such as chlorantraniliprole, have been widely applied in rice fields against rice borers [10]. Therefore, crop damage and high resistance emphasize the urgency for developing innovative control measures and resistance management strategies [7].

However, little is known about the neurotransmitter signaling systems in *C. suppressalis*. There were only some researches about octopamine, tyramine and acetylcholinesterases (AChEs) in *C. suppressalis*. Octopamine may play a role in mediating stress hormone effects on immune function via an α-adrenergic-like octopamine receptor [11] and is involved in the regulation of locomotion through a β-adrenergic-like octopamine receptor [12]. The molecular and pharmacological characterization of two tyramine receptors and two splicing variants of α_2_-adrenergic like octopamine receptors with different signaling properties have been reported in *C. suppressalis* [5, 13, 14]. In addition, RNA interference of AChE1 and AChE2 reveals their different contributions to motor ability and larval growth in *C. suppressalis* [15]. In an attempt to provide a more complete foundation for future molecular and physiological investigation of neurotransmitter signaling in *C. suppressalis*, we have initiated the characterization of neurochemical signaling systems using *de novo* sequencing of central nervous system transcriptome. In the present study, we have identified genes encoding enzymes and transporters with putative functions in neurotransmitter signaling systems. Since these genes are associated with biosynthesis and transport of neurotransmitters, they are prime targets of pesticides, and our work provides a valuable molecular resource for developing new effective and specific drugs for insect pest control.

## Results and discussion

### Illumina sequencing and unigenes assembly

**Fig. 1:**
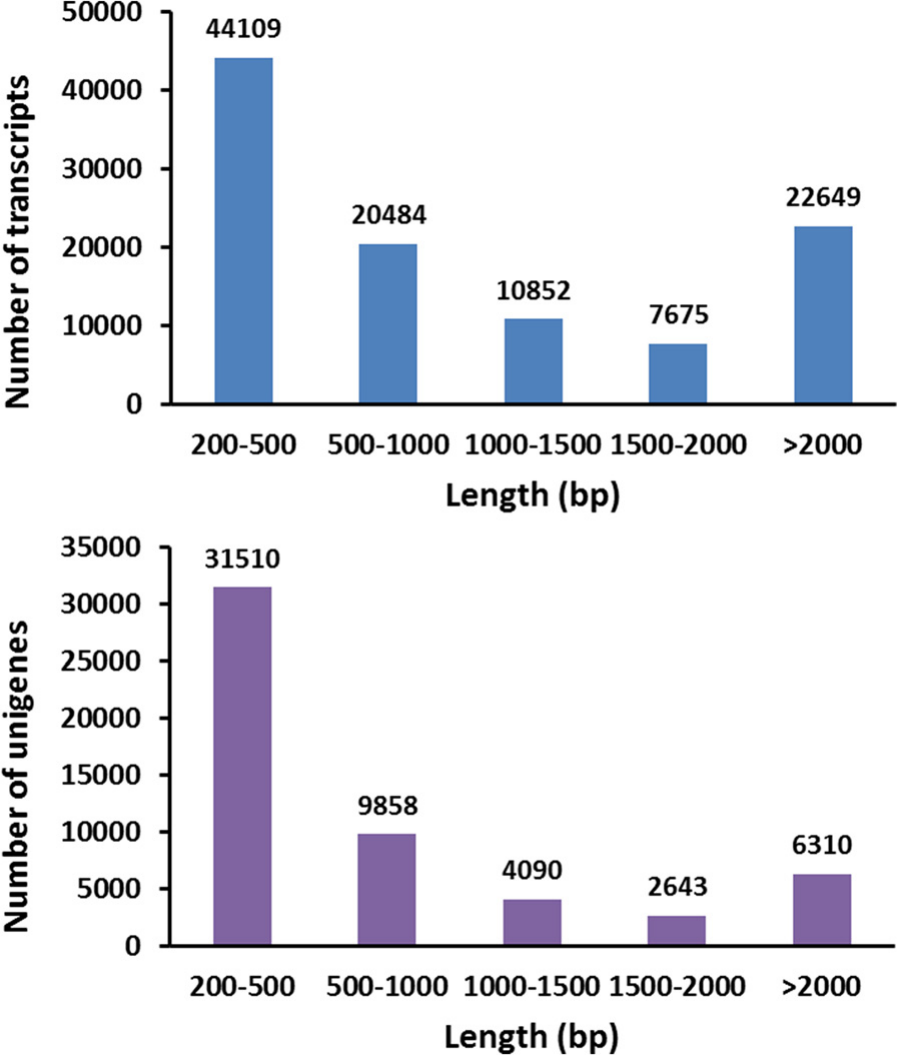
Length distribution of transcripts and unigenes in the *C. suppressalis* central nervous system transcriptome assembly

The transcriptomic sequence data were generated using a central nervous system cDNA library and Illumina HiSeq 2000 technology. For *C. suppressalis*, we acquired 142,051,094 bp raw reads from central nervous system transcriptome. After eliminating adapters, ambiguous nucleotides and low quality sequences, 138,063,130 bp clean reads remained, which accumulated to a total of 13.8 Gb with a GC percentage of 43.91 % (Table 1). Subsequently, *C. suppressalis* central nervous system transcriptome was *de novo* assembled using the short reads assembling program-Trinity [16]. Total clean base pairs yield 105,769 transcripts with an N50 length of 2,647 bp and an N90 length of 512 bp. These transcripts range from 201 to over 21,491 bp with an average size of 1,330 bp (Additional file 1). Among the transcripts, 44,109 (41.7 %) are between 200 bp and 500 bp long, and 21.4 % are over 2,000 bp (Fig. 1). After assembly of the transcripts into unigenes, 54,411 unigenes are obtained with an N50 length of 1,808 bp and an N90 length of 314 bp. These unigenes are from 201 to over 21,491 bp with an average size of 893 bp (Additional file 1). Among the assembled unigenes, 31,510 (41.7 %) are between 200 bp and 500 bp long, and 11.6 % are over 2,000 bp (Fig. 1). All sequences of the unigenes used in this study are provided in Additional file 2.

**Table 1:** The quality of *C. suppressalis* central nervous system unigene sequences and assembly

Raw reads (bp)	142,051,094
Clean reads (bp)	138,063,130
Clean base pairs (Gb)	13.8
Error (%)	0.03
Q20 (%)	98.00
Q30 (%)	93.03
GC (%)	43.91
Unigenes	54,411

### Functional annotation by searching against public databases

**Fig. 2:**
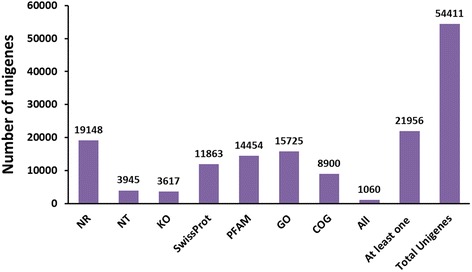
The number of unigenes annotated in public database searched

**Fig. 3:**
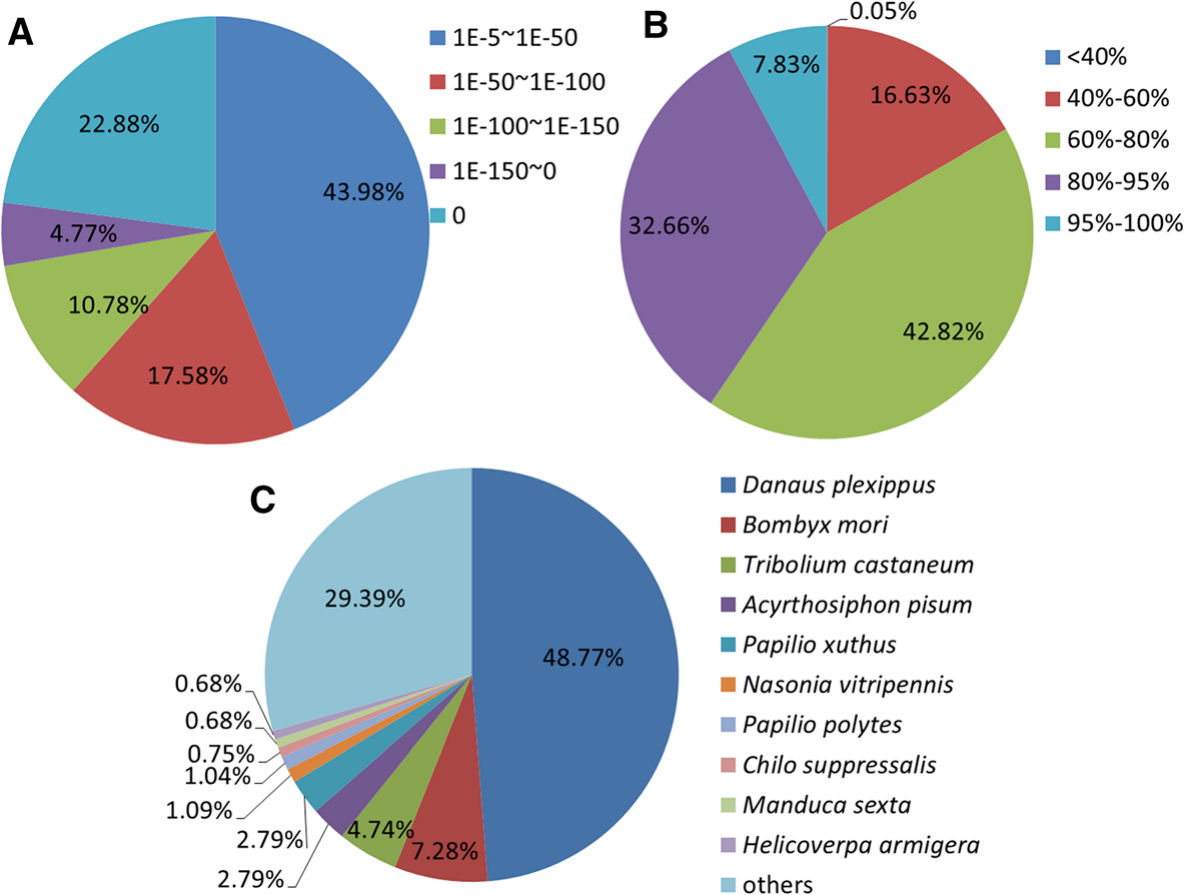
Characteristics of similarity search of unigenes against Nr database. (**a**) E-value distribution of BLAST hits for each unigene with a cut off E-value of 1.0E-5. (**b**) Similarity distribution of the top BLAST hits for each unigene. (**c**) Species distribution of the top BLAST hits for each unigene in Nr database

To validate and annotate the assembled unigenes, sequence similarity searches were conducted using the Nr, Nt, KEGG, SwissProt, PFAM, GO, and COG databases [17–19] with an E-value threshold of 10^−5^. The results indicated that out of 54,411 unigenes, 19,148 (35.2 %), 3,945 (7.3 %), 3,617 (6.6 %), 11,863 (21.8 %), 14,454 (26.6 %), 15,725 (28.9 %), and 8,900 (16.4 %) unigenes showed significant similarity to known proteins in Nr, Nt, KEGG, SwissProt, PFAM, GO, and COG databases, respectively. In addition, at 1,060 (1.9 %) or 21,956 (40.4 %) unigenes were annotated in all or at least one database (Fig. 2). The E-value distribution of the top hits in the Nr database revealed that 56.02 % of the mapped sequences showed significant homology matches (<1.0E-50) (Fig. 3a). The similarity distribution showed that 7.83 % of the sequences had > 95 % homology, followed by 75.48 % of the sequences with homology from 60 % to 95 %. Only 16.68 % of the sequences had homology lower than 60 % (Fig. 3b). Species specific distribution indicated that some of the *C. suppressalis* unigenes were homologous to those from more than one species, but most unigenes were homologous to Lepidoptera species with 12,013 hits among 19,148 BLASTn searches, including 9,339 (48.77 %) hits to *Danaus plexippus*, 1,394 (7.28 %) to *B. mori*, followed by 908 to *Tribolium castaneum*, and 535 to *Acyrthosiphon pisum*. The top 10 insect species that have significant BLASTn hits are shown in Fig. 3c.

### Gene Ontology (GO) and clusters of orthologous groups (COG) classification in *C. suppressalis* central nervous system transcriptome

Gene Ontology (GO) is an international standardized gene functional classification system which offers a dynamic-updated controlled vocabulary and a strictly defined concept to comprehensively describe the properties of genes and their products in any organism [20]. To further reveal their functions, GO assignments were used to classify *C. suppressalis* central nervous system unigenes. The 54,411 assembled unigenes were annotated into different functional groups according to Gene Ontology (GO) analysis. Some unigenes were annotated into more than one GO category. Of the 54,411 unigenes, 15,725 could be categorized into 54 functional groups (Additional file 3). The ‘cellular process’ and ‘metabolic process’ were most abundantly represented with 9,959 (66.33 %) and 8,894 (55.56 %) unigenes, respectively, within the ‘biological process’ GO ontology. In the ‘cellular components’ GO ontology the unigenes were mainly distributed in ‘cell’ (5,790 unigenes, 36.82 %) and ‘cell part’ (5,789 unigenes, 36.81 %). The GO analysis also showed that in the ‘molecular function’ ontology, 9,597 unigenes (61.03 %) have ‘binding’ functions and 7,450 (47.38 %) unigenes with ‘catalytic activity’ (Additional file 3).

The Clusters of Orthologous Groups (COG) is a database where the orthologous gene products are classified. Every protein in the COG database is assumed to be evolved from an ancestor protein, and the whole database is built on coding proteins with complete genome as well as systematic evolution relationships of bacteria, algae and eukaryotes [20]. All unigenes were aligned to the COG database to predict and classify potential functions. In total, 8,900 genes were assigned to the 25 COG classifications. Some unigenes were assigned to more than one COG category, thus a total of 9,920 sequences were assigned to 25 COG categories. Among the 25 COG categories, the cluster of ‘General function prediction only’ (2,276, 25.57 %) was the largest group, followed by ‘Signal transduction’ (1,170, 13.15 %), ‘Post-translational modification, protein turnover, chaperon’ (831, 9.34 %), ‘Transcription’ (574, 6.45 %), and ‘Function unknown’ (514, 5.78 %), whereas only a few unigenes were assigned to ‘Nuclear structure’ and ‘Cell motility’ (Additional file 4).

### Metabolic pathway analysis by KEGG

**Fig. 4:**
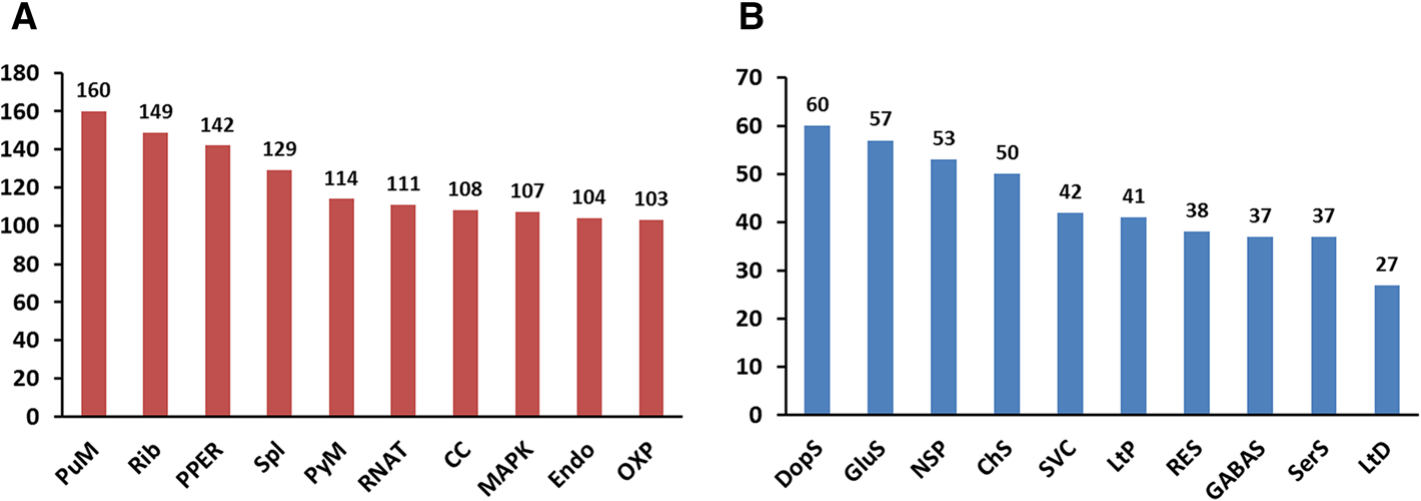
Distribution of unigenes among the KEGG pathways. (**a**) The top ten KEGG pathways with highest numbers of unigenes in *C. suppressalis* central nervous system. (**b**) The KEGG pathways in the pathway ‘Nervous system’. Abbreviation for pathways: Purine metabolism (PuM), Ribosome (Rib), Protein processing in endoplasmic reticulum (PPER), Spliceosome (Spl), Pyrimidine metabolism (PyM), RNA transport (RNAT), Cell cycle (CC), MAPK signaling pathway (MAPK), Endocytosis (Endo), Oxidative phosphorylation (OXP); Dopaminergic synapse (DopS), Glutamatergic synapse (GluS), Neurotrophin signaling pathway (NSP), Cholinergic synapse (ChS), Synaptic vesicle cycle (SVC), Long-term potentiation(LtP), Retrograde endocannabinoid signaling (RES), GABAergic synapse (GABAS), Serotonergic synapse (SerS), Long-term depression (LtD)

The Kyoto Encyclopedia of Genes and Genomes (KEGG) pathway database records the networks of molecular interactions in the cells, and variations of networks specific to particular organisms. Pathway-based analysis helps us to further understand the biological functions and interactions of genes [20]. In order to find out which biological pathways are active in *C. suppressalis* central nervous system, 54,411 unigenes were assigned to the reference pathways in KEGG. Consequently, 5,548 unigenes were mapped to 239 pathways. Among these pathways, ‘Purine metabolism’ (160 unigenes), ‘Ribosome’ (149 unigenes) and ‘Protein processing in endoplasmic reticulum’ (142 unigenes) (Fig. 4a) were the most common pathways in *C. suppressalis* central nervous system. Enrichment analysis is an effective way to identify the KEGG pathways that frequently occur in a tissue using the whole body transcriptome as background [21, 22]. In *C. suppressalis*, a total of 12 enriched KEGG pathways in central nervous system were identified (Additional file 5). Pathways like ‘Signal transduction’, ‘Amino acid metabolism’ and ‘Nervous system’ were enriched in *C. suppressalis* central nervous system. This is consistent with the principal function of insect central nervous system - uptake of neurotransmitters. In addition, ‘Transport and catabolism’ and ‘Endocrine system’ were also enriched. These transport-related pathways have been shown to be particularly crucial during the secretion of neurotransmitters and the formation of the action potential between the presynaptic terminal and the synaptic cleft. In the pathway ‘Nervous system’, 219 unigenes were assigned to 10 KEGG pathways, including ‘Dopaminergic synapse’ (60 unigenes), ‘Glutamatergic synapse’ (57), ‘Neurotrophin signaling pathway’ (53), ‘Cholinergic synapse’ (50), ‘Synaptic vesicle cycle’ (42), ‘Long-term potentiation’ (41), ‘Retrograde endocannabinoid signaling’ (38), ‘GABAergic synapse’ (37), ‘Serotonergic synapse’ (37), and ‘Long-term depression’ (27) (Fig. 4b).

### Candidate genes in *C. suppressalis* central nervous system with putative functions in neurotransmitter biosynthesis and transport

**Fig. 5:**
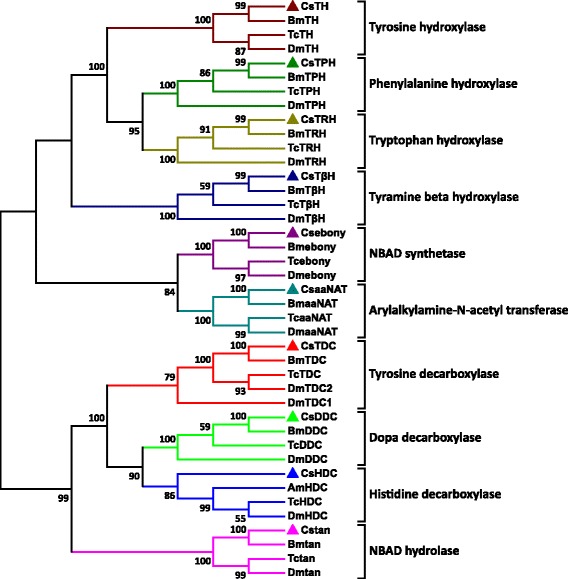
The phylogenetic analysis of enzymes involved in the biosynthesis pathway of biogenic amines in insects. Included are *C. suppressalis* (Cs), *B. mori* (Bm), *T. castaneum* (Tc), *D. melanogaster* (Dm), and *Apis mellifera* (Am). The accession numbers of the squences are available in Additional file 11. Neighbor-joining trees were constructed using MEGA 5 software with 1000-fold bootstrap re-sampling. The numbers at the nodes of the branches represent the level of bootstrap support for each branch

**Fig. 6:**
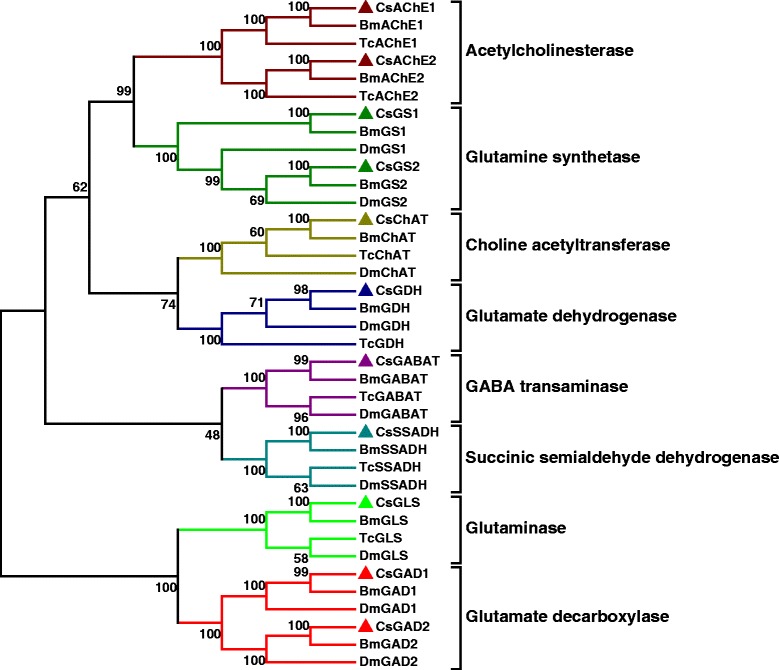
The phylogenetic analysis of enzymes involved in the biosynthesis pathway of acetylcholine, glutamate, and GABA in insects. Included are *C. suppressalis* (Cs), *B. mori* (Bm), *T. castaneum* (Tc), and *D. melanogaster* (Dm). The accession numbers of the squences are available in Additional file 11. Neighbor-joining trees were constructed using MEGA 5 software with 1000-fold bootstrap re-sampling. The numbers at the nodes of the branches represent the level of bootstrap support for each branch

**Fig. 7:**
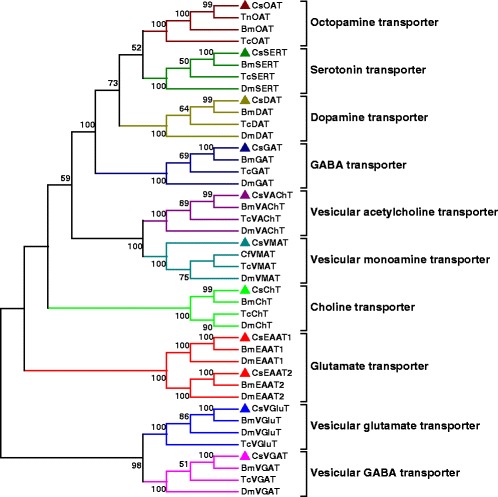
The phylogenetic analysis of transporters involved in neurotransmitter signaling systems in insects. Included are *C. suppressalis* (Cs), *T. ni* (Tn), *B. mori* (Bm), *T. castaneum* (Tc), *C. floridanus* (Cf), and *D. melanogaster* (Dm). The accession numbers of the squences are available in Additional file 11. Neighbor-joining trees were constructed using MEGA 5 software with 1000-fold bootstrap re-sampling. The numbers at the nodes of the branches represent the level of bootstrap support for each branch

**Fig. 8:**
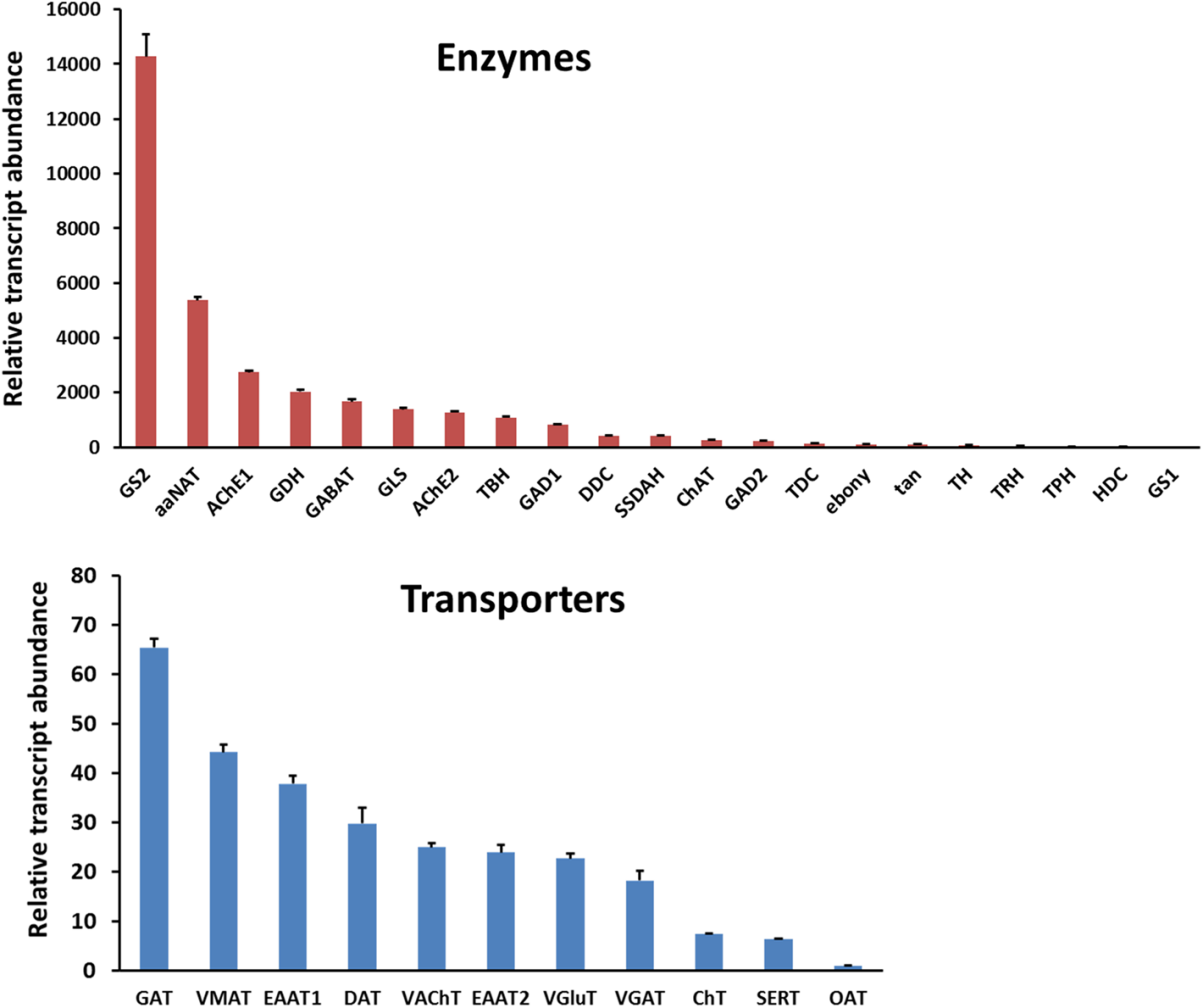
qRT-PCR results showing the relative transcript abundance of the unigenes encoding enzymes and transporters involved in the neurotransmitter signaling systems in the central nervous system of *C. suppressalis*

The overall enzymatic steps during neurotransmitter signaling system in *C. suppressalis* are likely to be similar to those in the fruit fly *Drosophila melanogaster* and other insect species, which include neurotransmitter biosynthesis, packaging, and recycling/degradation [1]. Therefore, we used deduced amino acid sequences of the genes associated with neurotransmitter biosynthesis and transport in *D. melanogaster* and other insects as queries for local BLAST to identify the candidate genes encoding enzymes and transporters in *C. suppressalis*. By homology search, we identified a number of unigenes in the *C. suppressalis* central nervous system putatively involved in biogenic aminergic signaling, acetylcholinergic signaling, glutamatergic signaling and GABAergic signaling, including unigenes putatively encoding 21 enzymes (Table 2) and 11 transporters (Table 3). In addition, we further confirmed the enzymes and transporters in *C. suppressalis* by comparing them with other known insect enzymes and transporters involved in neurotransmitter biosynthesis and transport using phylogenetic tree analysis (Figs. 5, 6 and 7). Their relative transcript abundances in the central nervous system were detected by qRT-PCR (Fig. 8). We further validated and characterized the expression levels of these genes by RT-PCR and qRT-PCR in various tissues and the results were discussed below.

**Table 2:** The enzymes involved in the biosynthesis pathway of neurotransmitters identified from *C. suppressalis* central nervous system transcriptome

Unigene	Gene	Accession No.	Length	ORF	Putative identification	Species	Accession No.	Score	E-value	Identity
comp54907_c0	TH	KP657623	2232	1683	tyrosine hydroxylase	*Spodoptera exigua*	AFG25778.1	1108	0	94 %
comp48842_c0	DDC	KP657625	2590	1440	dopa decarboxylase	*Antheraea pernyi*	AAR23825.1	906	0	89 %
comp56513_c0	ebony	KP657628	3245	2586	*ebony*	*Papilio xuthus*	BAE43845.2	1435	0	79 %
comp71328_c0	tan	KP657627	1994	1218	*tan* protein	*Bombyx mori*	NP_001170882.1	617	0	74 %
comp75401_c0	aaNAT	KP657626	1856	783	arylalkylamine N-acetyltransferase	*Bombyx mori*	NP_001073122.1	373	0	72 %
comp54468_c0	TDC	KP657629	2075	1878	aromatic amino acid decarboxylase	*Danaus plexippus*	EHJ72689.1	1075	0	85 %
comp60420_c0	TβH	KP657630	3530	1755	tyramine beta hydroxylase	*Bombyx mori*	NP_001243923.1	910	0	73 %
comp51351_c0	TRH	KP657632	1875	1590	tryptophan hydroxylase	*Bombyx mori*	XP_004929955.1	752	0	89 %
comp44225_c0	TPH	KP657631	2087	1365	phenylalanine hydroxylase	*Papilio xuthus*	BAE66652.1	851	0	88 %
comp52601_c0	HDC	KP657633	2475	1773	histidine decarboxylase	*Danaus plexippus*	EHJ77965.1	1043	0	85 %
comp36379_c0	ChAT	KP657655	833		choline acetyltransferase	*Bombyx mori*	BAO23491.1	355	4E-114	83 %
comp63891_c2	AChE1	KP657634	5043	2085	acetylcholinesterase 1	*Chilo suppressalis*	ABO38111.1	1443	0	99 %
comp66146_c1	AChE2	KP657635	2255	1917	acetylcholinesterase 2	*Chilo suppressalis*	ABR24230.1	1334	0	100 %
comp57261_c0	GLS	KP657636	3061	1932	putative glutaminase	*Danaus plexippus*	EHJ71111.1	1170	0	87 %
comp56484_c0	GS1	KP657637	1379	1215	glutamine synthetase 1	*Bombyx mori*	XP_004930366.1	647	0	75 %
comp54696_c0	GS2	KP657638	5657	1143	glutamine synthetase 2	*Papilio xuthus*	BAM17922.1	706	0	93 %
comp52410_c0	GDH	KP657639	2318	1665	glutamate dehydrogenase	*Papilio polytes*	BAM20330.1	1115	0	95 %
comp63917_c0	GAD1	KP657640	3698	1623	glutamate decarboxylase-like	*Bombyx mori*	XP_004925034.1	984	0	93 %
comp56481_c0	GAD2	KP657641	2518	1530	black	*Biston betularia*	AEP43793.2	941	0	86 %
comp52445_c0	GABAT	KP657642	3275	1482	4-aminobutyrate aminotransferase	*Danaus plexippus*	EHJ72994.1	771	0	74 %
comp58969_c0	SSADH	KP657643	2049	1521	succinate-semialdehyde dehydrogenase	*Bombyx mori*	XP_004932642.1	833	0	77 %

**Table 3:** The transporters involved in the neurotransmitter signaling pathways identified from *C. suppressalis* central nervous system transcriptome

Unigene	Gene	Accession No.	Length	ORF	Putative identification	Species	Accession No.	Score	E-value	Identity
comp66192_c1	DAT	KP657644	2448	1839	dopamine transporter	*Bombyx mori*	NP_001037362.1	1076	0	90 %
comp62798_c0	OAT	KP657645	2908	2250	high-affinity octopamine transporter	*Ostrinia nubilalis*	AAZ08592.2	1340	0	91 %
comp64226_c0	SERT	KP657646	3079	1782	serotonin transporter	*Bombyx mori*	NP_001037436.1	1071	0	90 %
comp61773_c0	VMAT	KP657647	1982	1458	synaptic vesicular amine transporter	*Camponotus floridanus*	EFN70897.1	625	0	73 %
comp60635_c0	ChT	KP657648	4324	1782	high-affinity choline transporter	*Trichoplusia ni*	AAT88074.1	1091	0	93 %
comp63510_c0	VAChT	KP657649	4635	1863	vesicular acetylcholine transporter-like	*Bombyx mori*	NP_001275599.1	1070	0	87 %
comp71360_c0	EAAT1	KP657650	1655	1452	excitatory amino acid transporter 1	*Trichoplusia ni*	AAB84380.1	839	0	86 %
comp63601_c1	EAAT2	KP657651	2956	1623	excitatory amino acid transporter 2	*Bombyx mori*	NP_001240825.1	741	0	83 %
comp52964_c0	VGluT	KP657652	2707	1782	vesicular glutamate transporter	*Bombyx mori*	XP_004925576.1	972	0	85 %
comp65336_c0	GAT	KP657653	5728	1824	high affinity GABA transporter	*Trichoplusia ni*	AAF70819.1	1179	0	96 %
comp62581_c0	VGAT	KP657654	2512	1617	vesicular GABA transporter	*Danaus plexippus*	EHJ77951.1	947	0	88 %

### Biogenic aminergic signaling

Biogenic amines are one class of signaling molecules used by both vertebrate and invertebrate nervous systems, and they play a key role in regulating and modulating various physiological and behavioral processes. In insects, five biogenic amines are generally recognized: dopamine, octopamine, tyramine, serotonin and histamine [23].

### Dopaminergic signaling

**Fig. 9:**
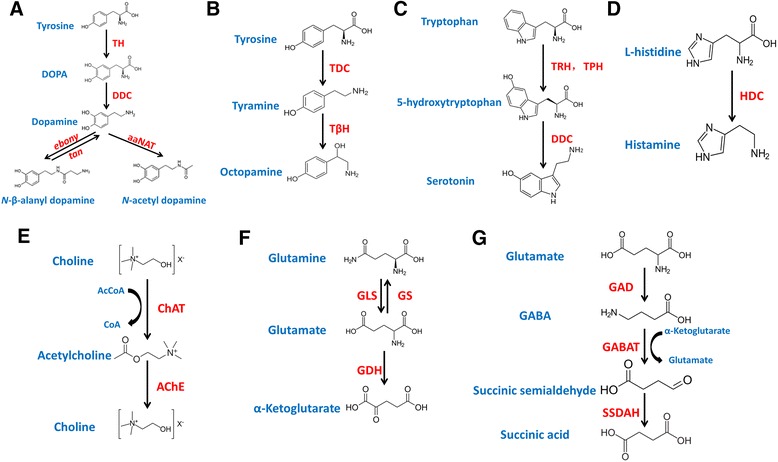
The biosynthesis pathway of neurotransmitters in insects. **a** The biosynthesis pathway of dopamine. **b** The biosynthesis pathway of tyramine and octopamine. **c** The biosynthesis pathway of serotonin. (**d**) The biosynthesis pathway of histamine. **e** The biosynthesis pathway of acetylcholine. **f** The biosynthesis pathway of glutamate. **g** The biosynthesis pathway of GABA

Dopamine is found at a relatively high level throughout the insect nervous system and is the most abundant monoamine present [24]. In insects, dopamine modulates various aspects of behavior such as locomotor activity [25], decision-making [26], phase change [27], copulation persistence [28], sucrose acceptance [29], learning and memory [30], and is also a precursor of melanin. To produce dopamine, tyrosine is first converted to L-3, 4-dihydroxyphenylalanine (L-DOPA) via the action of tyrosine hydroxylase (TH), which is then converted to dopamine by DOPA decarboxylase (DDC) [31]. Dopamine is also utilized by NBAD synthase (*ebony*) and arylalkylamine N-acetyl transferase (aaNAT) to produce pigments other than melanin. NBAD hydrolase (*tan*) catalyzes the reaction in the opposite direction to *ebony*, increasing dopamine concentration [32] (Fig. 9a). Dopamine is released as a neurotransmitter from synaptic vesicles via exocytosis at presynaptic terminal [33]. Subsequently, the reuptake of dopamine from the synaptic cleft can be accomplished through its interaction with plasma membrane monoamine transporter - dopamine transporter (DAT) [34]. Next, the refluent dopamine can be transported to synaptic vesicles for storage through vesicular monoamine transporter(VMAT), which is also likely to function as a vesicular transporter for the storage of serotonin and octopamine [33, 35].

**Fig. 10:**
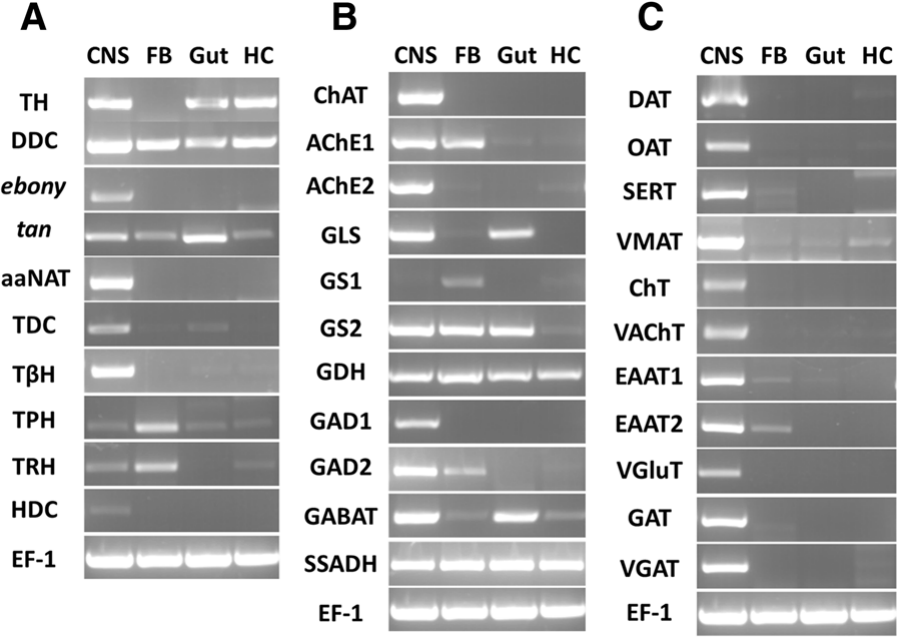
RT-PCR results showing the relative expression of the *C. suppressalis* neurotransmitter-related genes in various tissues. **a** The relative expression of the enzymes involved in the biosynthesis pathway of biogenic amines in various tissues; **b** The relative expression of the enzymes involved in the biosynthesis pathway of acetylcholine, glutamate, and GABA in various tissues; **c** The relative expression of the transporters involved in the neurotransmitter signaling systems in various tissues. EF-1 was used as internal reference gene to test the integrity of each cDNA templates; the similar intensity of EF-1 bands between various tissues indicate the use of equal template concentrations. CNS, central nervous system; FB, fat body; Gut, gut; HC, hemocytes

**Fig. 11:**
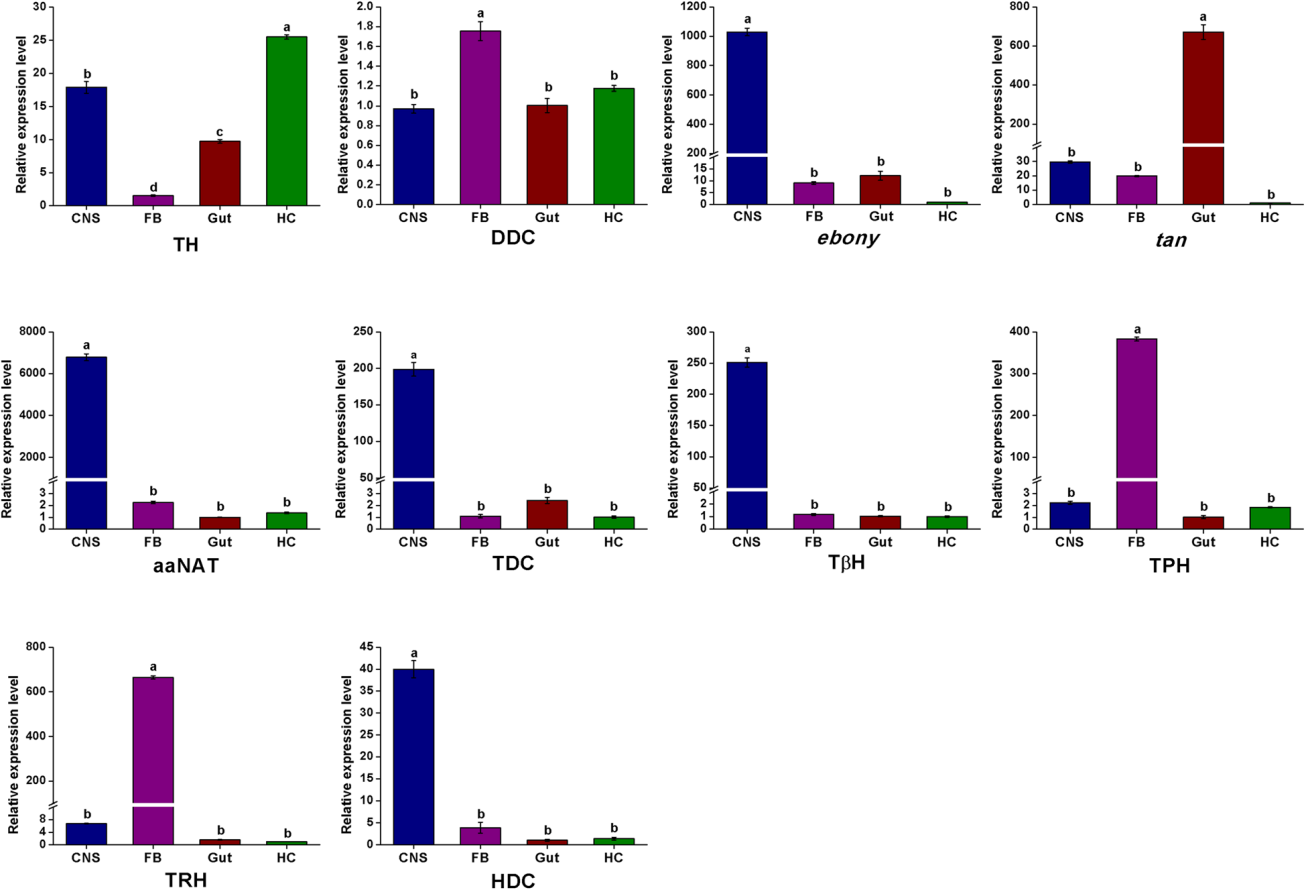
qRT-PCR results showing the relative expression levels of the enzymes involved in the biosynthesis pathway of biogenic amines in various tissues in *C. suppressalis*. The standard error is represented by the error bar, and the different letters above each bar denote significant differences (p < 0.05)

To elucidate the molecular basis of the dopaminergic signaling system, we identified dopamine-related genes involved in biosynthesis, signal transduction, and reuptake in *C. suppressalis* central nervous system transcriptome. We obtained five enzymes and two transporters, TH, DDC, aaNAT, *ebony*, *tan*, DAT and VMAT. Interestingly, TH has two splicing variants, the long isoform has an open reading frame (ORF) of 1,683 bp and the deduced amino acid sequence shows 94 % identity to TH of *Spodoptera exigua* (Protein ID: AFG25778.1), whereas the short isoform has an ORF of 1,527 bp. The alternative splicing mechanism has also been found in *Mythimna separat*a and *D. melanogaster* [36]. Multiple sequence alignment showed that the alternative splicing domains were conserved in THs (Additional file 6). DDC contains an open reading frame of 1,440 bp and its amino acid sequence is 89 % identity to DDC of *Antheraea pernyi* (Protein ID: AAR23825.1). Comparison of *C. suppressalis* aaNAT with *B. mori* aaNAT (Protein ID: NP_001073122.1) revealed 72 % identity in amino acid sequences. Two unigenes encoding ebony and tan proteins in *C. suppressalis* show 79 % identity in amino acid sequence to ebony of *Papilio xuthus* (Protein ID: BAE43845.2 ) and 74 % identity to tan protein of *B. mori* (Protein ID: NP_001170882.1), respectively (Table 2). In addition, two transporters, DAT and VMAT, show 90 % and 73 % identities in amino acid sequences with DAT of *B. mori* (Protein ID: NP_001037362.1) and VMAT of *Camponotus floridanus* (Protein ID: EFN70897.1), respectively (Table 3). The RT-PCR and qRT-PCR results revealed that TH and DDC were highly expressed in the hemocytes in addition to central nervous system (Figs. 10a and 11). Exogenous stimuli could induce expression of TH and DDC, suggesting that dopamine may be an important molecule bridging the nervous system and immune system [37]. The expression level of aaNAT and *ebony* in the central nervous system was highest, while *tan* was expressed at the highest level in the gut (Figs. 10a and 11). Moreover, the RT-PCR and qRT-PCR results showed that two transporters were expressed at significantly high levels in the central nervous system (Figs. 10c and 13), indicating that these two transporters are likely to play an vital role in regulating the storage and release of dopamine in the nervous system [35].

### Octopaminergic and tyraminergic signaling

Octopamine is a biogenic amine with a widespread distribution in the insect central nervous system [38, 39]. The structures of octopamine and tyramine differ only in the respective presence or absence of a hydroxyl group at β-position in their side chains [40]. Octopamine and tyramine are considered to be the invertebrate counterparts of the vertebrate adrenergic transmitters, and the two phenolamines are the only biogenic amines whose physiological significance is presumably restricted to invertebrates [5, 13, 41, 42]. They regulate various physiological functions in insects, such as addiction, circadian rhythms, endocrine secretion, aggression, egg-laying, food-seeking, locomotion and decision-making [12, 43]. For the production of octopamine, tyrosine is converted by tyrosine decarboxylase (TDC) to tyramine, which in turn is converted to octopamine via the action of tyramine beta hydroxylase (TβH) [31] (Fig. 9b). Octopamine and tyramine can be released from synaptic vesicles into synaptic cleft, then octopamine transporter acts in the reuptake system for both transmitters in the neurons involved in octopaminergic/tyraminergic signaling [24]. As mentioned above, vesicular monoamine transporter (VMAT) can function as a vesicular transporter for the storage of octopamine and tyramine [33].

To investigate the molecular basis of the octopaminergic/tyraminergic signaling system, we identified genes participating in octopamine/tyramine biosynthesis, signal transduction, and reuptake in *C. suppressalis* central nervous system transcriptome. We obtained two enzymes and one transporter, including TDC, TβH and OAT. TDC contains an open reading frame of 1,878 bp and the deduced amino acid sequence is 85 % identity to TDC of *D. plexippus* (Protein ID: EHJ72689.1). The unigene with an open reading frame of 1,755 bp encodes a TβH with 73 % identity to TβH of *B. mori* (Protein ID: NP_001243923.1) (Table 2). In addition, the transporter OAT shows 91 % identity with OAT of *Ostrinia nubilalis* (Protein ID: AAZ08592.2) (Table 3). Unfortunately, no octopamine transporter has thus far been characterized from *D. melanogaster*. This is puzzling because both compounds must be removed following release. The only biogenic amine transporters found in *Drosophila*, DAT and SERT, have pharmacological features that are not tuned to take up these major neuroactive compounds. Although they are not for octopamine/tyramine uptake, these transporters are of great interest because they are prime targets for pharmacological compounds [41]. The RT-PCR and qRT-PCR results revealed that TDC, TβH and OAT were highly expressed in the central nervous system (Figs. 10a, c and 11, 13), suggesting that these genes are likely to play an important role in biosynthesis and transport of octopamine and tyramine in *C. suppressalis* central nervous system. Interestingly, octopamine also seems to play a role in the immune system of invertebrates, which resembles the innate immune system in humans, as its concentration in the hemolymph increases during an immune challenge [44]. In addition, a concentration-sensitive α-adrenergic-like octopamine receptor is found on insect immune cells and plays a possible role in mediating stress hormone effects on immune function [11].

### Serotoninergic signaling

The biogenic amine serotonin, or 5-hydroxytryptamine (5-HT), is widely distributed in animals. It acts through multiple receptors to modulate many complex behaviors in vertebrates and invertebrates [45]. In insects, serotonin signaling controls nutrition, heart rate, secretory processes, feeding, gut contraction, development, circadian rhythms, sleep, aggression, behavioral gregarization, phototactic behavior, learning, and memory [46, 47]. For the production of serotonin, tryptophan is converted to 5-hydroxytryptophan via either phenylalanine hydroxylase (TPH) or tryptophan hydroxylase (TRH), which in turn is converted to serotonin by DDC [31] (Fig. 9c). Serotonin is released from secretory vesicles into synaptic cleft like dopamine and octopamine, and serotonin transporter (SERT) can mediate reuptake as the primary extracellular mechanism for clearing of released serotonin, and SERT-mediated reuptake is critical for maintaining a high intracellular serotonin pool [48]. In *D. melanogaster*, tryptophan hydroxylase is encoded by two genes, dTPH and dTRH [49]. DDC is involved in the biosynthesis of not only serotonin but also dopamine, and thus DDC is expressed in serotoninergic and dopaminergic neurons in the central nervous system [50].

To illustrate the molecular basis of the serotoninergic signaling system, we identified serotonin-related genes associated with serotonin biosynthesis, signal transduction and reuptake in *C. suppressalis* central nervous system transcriptome. We obtained two enzymes and one transporter, including TPH, TRH and SERT. TPH contains an open reading frame of 1,365 bp with 88 % identity in amino acid sequence to TPH of *P. xuthus* (Protein ID: BAE66652.1). The unigene with an open reading frame of 1,590 bp encodes a TRH with 89 % identity to TRH of *B. mori* (Protein ID: XP_004929955.1) (Table 2). Moreover, the transporter SERT shows 90 % identity in amino acid sequence with SERT of *B. mori* (Protein ID: NP_001037436.1) (Table 3). The RT-PCR and qRT-PCR results showed that TPH and TRH were highly expressed in the fat body (Figs. 10a and 11), while the transporter SERT was specifically expressed in the central nervous system (Figs. 10c and 13). It is interesting that serotonin synthesis and storage are also found in immune cells, and serotonin participates in innate immune response and adaptive immunity. Thus, serotonin also plays an important role in immune signaling outside of the central nervous system [51].

### Histaminergic signaling

The biogenic amine histamine (HA) is among the first compounds recognized as a messenger molecule in intercellular communication [52]. In vertebrates, it functions as a transmitter in the brain as well as a transmitter, hormone and mediator in peripheral systems. In the periphery, histamine is responsible for different actions such as the contraction of smooth muscle, capillary dilation or stimulation of gastric secretion [53]. In invertebrates, histamine has various roles in neurotransmission in the brain, such as olfaction in crustaceans and photoreception in various arthropods, as well as in mechanoreception [54]. In arthropods, it was reported that histamine increases chloride conductance [55] and that its receptors are members of the ligand-gated chloride channel family. In *D. melanogaster*, histamine can modulate temperature preference behaviors [56] and regulate wake-promoting signals [57]. Immunohistochemical studies indicated the presence of histamine in a variety of neuron types in the brain and optic lobes, as well as in the ganglia of the ventral nerve cord of several insect species [58]. To produce histamine, the amino acid histidine is decarboxylated via a reaction catalyzed by histidine decarboxylase (HDC) [54] (Fig. 9d).

In order to clarify the molecular basis of the histaminergic signaling system, we identified histamine-related genes responsible for biosynthesis, signal transduction and reuptake in *C. suppressalis* central nervous system transcriptome. We only obtained one enzyme, HDC. Comparison of the deduced amino acid sequences of *C. suppressalis* HDC with other HDCs, indicated that *Chilo* HDC is most similar to the other known insect HDCs (89 % identical to *Danaus* HDC, 68 % to *Apis* HDC, 66 % to *Drosophila* HDC, and 64 % to *Tribolium* HDC). The phylogenetic analysis of aromatic amino acid hydroxylases using the amino acid sequence of the *Chilo* HDC and various other aromatic amino acid hydroxylases also indicated that *Chilo* HDC is closely related to the insect HDC proteins (Fig. 5). The RT-PCR and qRT-PCR results demonstrated that HDC was highly expressed in the central nervous system (Figs. 10a and 11), indicating that the production of histamine is likely to occur mainly in the central nervous system. Unfortunately, the transporter that mediates histamine reuptake has not been fully characterized so far [59], but there is evidence that organic cation transporter (OCT) can function as a histamine transporter as well [60].

### Acetylcholinergic signaling

Acetylcholine is the most abundant neurotransmitter in the brain of insects, particularly in the sensory pathways and specifically in the olfactory system. Acetylcholine is thought to be the major excitatory neurotransmitter in the central nervous system of insects as attested in honeybees, flies, grasshoppers, and locusts [61]. The acetylcholinergic system is one of the excitatory pathways participating in the parasympathicus, sympathicus, and the central nervous system using acetylcholine as a neurotransmitter [3]. Acetylcholine is synthesized from acetyl-CoA and choline via the action of the enzyme choline O-acetyltransferase (ChAT). Following the synthesis of acetylcholine, acetylcholine generated in neurons is antiported by protons into secretory vesicles using vesicular acetylcholine transporter (VAChT). Upon its release from nerve terminal, triggered by an action potential, acetylcholine acts on its target tissues through two distinct receptor types, including nicotinic acetylcholine receptors (nAChRs) and muscarinic acetylcholine receptors (mAChRs) [62, 63]. Acetylcholine is a chemically stable compound that can persist for a long time after spreading into the synaptic cleft and spontaneous elimination is slow due to the quaternary ammonium atom in the choline moiety [3]. For this reason, the enzyme acetylcholinesterase (AChE) is just present in the synaptic cleft to quickly terminate the signal, and AChE hydrolyzes acetylcholine into acetic acid and choline [64]. While choline is transported from the synaptic cleft using choline transporter (ChT) back to the cytosol, the choline and acetic acid produced are then taken up and recycled by cholinergic neurons as precursors in new acetylcholine synthesis [65, 66] (Fig. 9e).

**Fig. 12:**
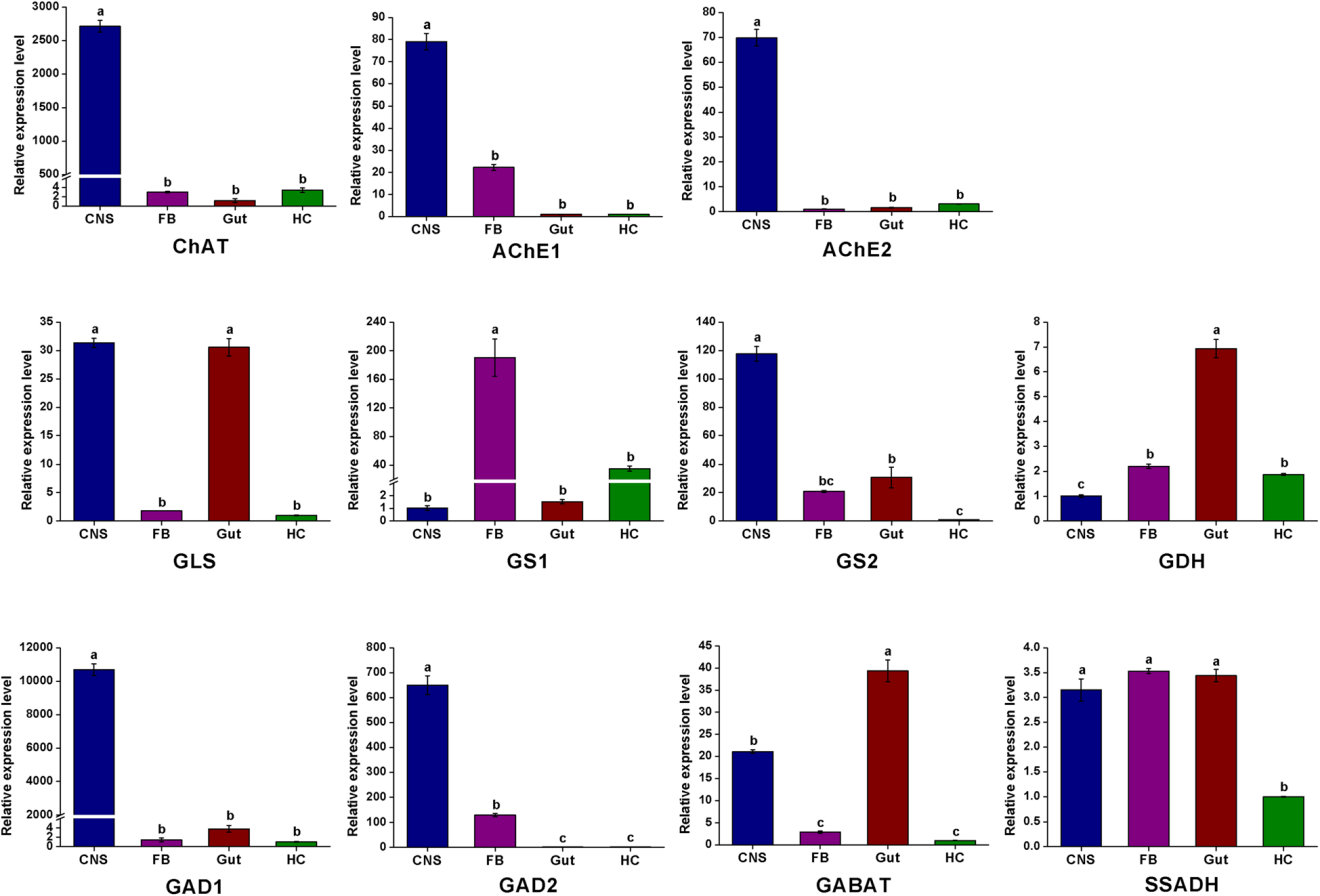
qRT-PCR results showing the relative expression levels of the enzymes involved in the biosynthesis pathway of acetylcholine, glutamate, and GABA in various tissues in *C. suppressalis*. The standard error is represented by the error bar, and the different letters above each bar denote significant differences (p < 0.05)

**Fig. 13:**
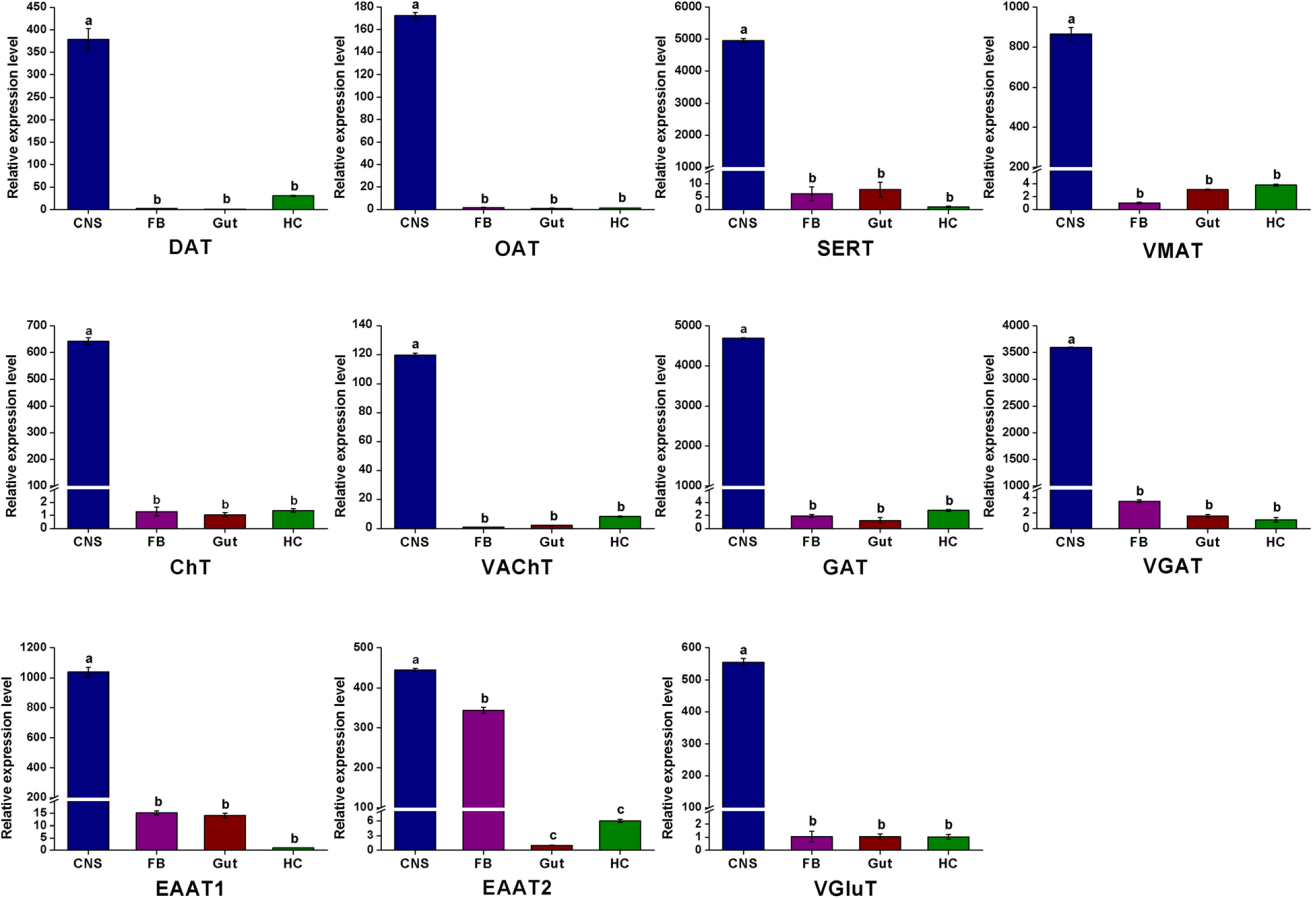
qRT-PCR results showing the relative expression levels of the transporters involved in the neurotransmitter signaling systems in various tissues in *C. suppressalis*. The standard error is represented by the error bar, and the different letters above each bar denote significant differences (p < 0.05)

To elucidate the molecular basis of the acetylcholinergic signaling system, we identified acetylcholine-related genes involved in biosynthesis, signal transduction, and reuptake in *C. suppressalis* central nervous system transcriptome. We obtained three enzymes and two transporters, including ChAT, AChE1, AChE2, VAChT and ChT. Partial ChAT sequence containing 833 bp shows 83 % identity in amino acid sequence with ChAT of *B. mori* (Protein ID: BAO23491.1). Two unigenes encoding AChE1 and AChE2 in *C. suppressalis* transcriptome show 100 % identity in amino acid sequences with the published AChE1 and AChE2 of *C. suppressalis* (Protein ID: ABO38111.1 and ABR24230.1, respectively) (Table 2). Multiple sequence alignment of acetylcholinesterases revealed that choline binding sites and active site triad residues were conserved in AChEs (Additional file 7). AChEs have been specialized as the main catalytic enzymes and become very effective targets for both organophosphorus and carbamate insecticides in different insect species [67–69]. In addition, two transporters ChT and VAChT show 93 % and 87 % identity in amino acid sequences with ChT of *Trichoplusia ni* (Protein ID: AAT88074.1) and VAChT of *B. mori* (Protein ID: NP_001275599.1), respectively (Table 3). The RT-PCR and qRT-PCR results revealed that ChAT, AChE1 and AChE2 were all highly expressed in the central nervous system (Figs. 10b and 12). ChAT with higher activity is localized in the cytosol near neurosynapses as a soluble molecule, and ChAT can also be found as a membrane bound protein [65]. AChEs are essential enzymes at the synapses of cholinergic neurons in the central and peripheral nervous systems, to catalyze the hydrolysis of the neurotransmitter acetylcholine, thus terminating neurotransmission [70]. Additionally, the RT-PCR and qRT-PCR results revealed that ChT and VAChT were significantly expressed in the central nervous system (Fig. 10c and 13), indicating that  the two transporters may play a major role in neurotransmitter uptake and neurotransmission. Interestingly, acetylcholine and acetylcholine receptors are known to be present on many cell types, including endothelial cells and cells of the immune system, suggesting that there exists a connection between the immune system and the nervous system [71].

### Glutamatergic signaling

Glutamate is a major neurotransmitter in both vertebrates and invertebrates. Glutamate acts as an excitatory neurotransmitter in vertebrates, whereas it functions as both an excitatory and an inhibitory neurotransmitter in invertebrates. Two closely related amino acids, γ-aminobutyric acid and glutamate, play an important role as the major inhibitory neurotransmitters in invertebrates. Glutamatergic inhibitory neurotransmission is mediated by pentameric glutamate-gated chloride channels in the invertebrate nervous system [72]. In arthropods, glutamate participates in regulating multiple physiological processes such as neuron sensibility modulation [73], juvenile hormone synthesis [74], control of rest/arousal neurons [75], olfactory memory [76], locomotion and feeding [77]. Glutamate is synthesized from glutamine via the action of the enzyme glutaminase (GLS). Within neurons, packaging of this neurotransmitter is achieved via vesicular glutamate transporter (VGluT). Glutamate released from synaptic vesicles acts on target tissues via a number of glutamate receptors. Following its release, the actions of glutamate are terminated by its reuptake via excitatory amino acid transporters (EAATs) located primarily in glia, and its subsequent conversion back to glutamine via the action of glutamine synthetase (GS), or degradation into α-ketoglutarate via the action of glutamate dehydrogenase (GDH) [1] (Fig. 9f).

To illustrate the molecular basis of the glutamatergic signaling system, glutamate-related genes involved in biosynthesis, signal transduction, and reuptake were found in *C. suppressalis* central nervous system transcriptome. We found four enzymes and three transporters, including GLS, GS1, GS2, GDH, VGluT, EAAT1 and EAAT2. Comparison of *C. suppressalis* GLS (643 amino acids) with *D. plexippus* GLS (Protein ID: EHJ71111.1) revealed 87 % identity between the two proteins. Two unigenes encoding GS1 and GS2 in *C. suppressalis* transcriptome show 75 % and 93 % identities amino acid sequences with GS1 of *B.mori* (Protein ID: XP_004930366.1) and GS2 of *P. xuthus* (Protein ID: BAM17922.1), respectively (Table 2). Amino acid sequence alignment of glutamine synthetases suggested that the residues involved in binding of glutamate, ATP, and ammonia were conserved in GSs (Additional file 8). GDH contains an open reading frame of 1,665 bp with 95 % identity in amino acid sequence to GDH of *Papilio polytes* (Protein ID: BAM20330.1) (Table 2). VGluT with an open reading frame of 1,782 bp shows 85 % identity in amino acid sequence to *B.mori* VGluT (Protein ID: XP_004925576.1). Moreover, two excitatory amino acid transporters function as glutamate transporters, two unigenes encoding EAAT1 and EAAT2 show 86 % and 83 % identities in amino acid sequences with EAAT1 of *T. ni* (Protein ID: AAB84380.1) and EAAT2 of *B.mori* (Protein ID: NP_001240825.1), respectively (Table 3). Multiple sequence alignment of excitatory amino acid transporters indicated that the amino acid sequences of the two types of EAATs were conserved (Additional file 9). The RT-PCR and qRT-PCR results revealed that GLS was highly expressed in the central nervous system and gut, GS1 was highly expressed in fat body, GS2 was highly expressed in the central nervous system, while GDH was highly expressed in gut (Figs. 10b and 12). In addition, the RT-PCR and qRT-PCR results revealed that VGluT and EAAT1 were specifically expressed in the central nervous system, whereas EAAT2 was highly expressed in both central nervous system and fat body (Figs. 10c and 13). In *D. melanogaster*, EAAT1 was expressed in a population of neurons, located between the lamina and medulla neuropils of the optic lobes, to address glial processes that closely follow the motor axons up to the neuromuscular junction. EAAT2 was expressed in the central and peripheral nervous systems to regulate selective olfactory and gustatory functions [78].

### GABAergic signaling

Neurons communicate with each other via signaling molecules. In vertebrate fast neurotransmission, γ-aminobutyric acid (GABA) acts as an inhibitory neurotransmitter. The precursor of GABA is glutamate. It is interesting that these two closely related amino acids share an inhibitory neurotransmitter role in invertebrates [79]. For insects, GABA is reported to modulate various physiological behaviors such as copulation persistence [28], feeding restraint [80], locomotion [81], sleep [82], circadian clock [83], response to alcohol [84], and olfactory memory [85]. GABA is synthesized from glutamate via the actions of the enzyme glutamic acid decarboxylase (GAD). Once synthesized, vesicular GABA transporter (VGAT) packages GABA into synaptic vesicles. GABA is released into synaptic cleft from the presynaptic neurons by depolarization triggered by the action potential. The released GABA traverses the synaptic cleft and binds to ionotropic receptors localized in the postsynaptic membrane to provoke an electrical change in the postsynaptic neurons. GABA also activates GPCRs to elicit intracellular signal transduction. Then, the actions of GABA are terminated by its reuptake via GABA transporter (GAT) and its subsequent conversion back to glutamate via the action of GABA transaminase (GABAT), or degradation via the enzyme succinic semialdehyde dehydrogenase (SSADH) [1, 86, 87] (Fig. 9g).

In *C. suppressalis* central nervous system transcriptome, GABA-related genes responsible for biosynthesis, signal transduction, and reuptake were identified. We found GAD1, GAD2, GABAT, SSADH, VGAT and GAT. GABA is synthesized by two isoforms of the pyridoxal 5’-phosphate-dependent enzyme glutamic acid decarboxylase (GAD1 and GAD2) [88]. Two unigenes encoding GAD1 and GAD2 in *C. suppressalis* transcriptome show 83 % and 86 % identities in amino acid sequences with GAD1 of *B.mori* (Protein ID: XP_004925034.1) and GAD2 of *Biston betularia* (Protein ID: AEP43793.2), respectively (Table 2). Multiple sequence alignment of glutamate decarboxylase showed that the proposed substrate binding domain, the ‘decarboxylation’ domain, active site residues, and functional residues were conserved in GADs (Additional file 10). GABAT with an open reading frame of 1,482 bp shows 74 % identity in amino acid sequence to *D. plexippus* GABAT (Protein ID: EHJ72994.1). SSADH contains an open reading frame of 1,521 bp with 77 % identity in amino acid sequence to SSADH of *B.mori* (Protein ID: XP_004932642.1) (Table 2). In addition, the two transporters VGAT and GAT show 88 % and 96 % identities in amino acid sequences with VGAT of *D. plexippus* (Protein ID: AAT88074.1) and GAT of *T. ni* (Protein ID: AAF70819.1), respectively (Table 3). The RT-PCR and qRT-PCR results showed that both GAD1 and GAD2 were specifically expressed in the central nervous system, GABAT was highly expressed in the central nervous system and gut, while SSADH was not significantly different at expression levels in various tissues (Figs. 10b and 12). Furthermore, the two transporters VGAT and GAT were significantly expressed in the central nervous system (Figs. 10c and 13).

## Conclusions

**Fig. 14:**
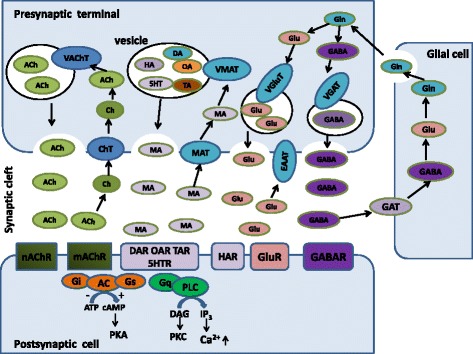
Putative neurotransmitter signaling pathways in insects. ACh, acetylcholine; Ch, choline; ChT, choline transporter; VAChT, vesicular acetylcholine transporter; nAChR, nicotinic acetylcholine receptor; mAChR, muscarinic acetylcholine receptor; DA, dopamine; OA, octopamine; TA, tyramine; 5HT, serotonin; HA, histamine; MA, monoamine; MAT, monoamine transporter; VMAT, vesicular monoamine transporter; DAR, dopamine receptor; OAR, octopamine receptor; TAR, tyramine receptor; 5HTR, serotonin receptor; HAR, histamine receptor; Glu, glutamate; Gln, glutamine; EAAT, excitatory amino acid transporter; VGluT, vesicular glutamate transporter; GluR, glutamate receptor; GABA, γ-aminobutyric acid; GAT, GABA transporter; VGAT, vesicular GABA transporter; GABAR, GABA receptor; AC, adenylyl cyclase; DAG, diacylglycerol; IP3, 1,4,5-trisphosphate; PKA, protein kinase A; PKC: protein kinase C; PLC, phospholipase C; ATP, adenosine triphosphate; cAMP, cyclic adenosine monophosphate

The rice striped stem borer *C. suppressalis* is a destructive rice pest in China. In recent years, the damage caused by *C. suppressalis* has increased dramatically in China and has posed a severe threat to high and stable crop yields through changes in the rice cultivation system and the popularization of hybrid rice. To date, control of this insect still relies mainly on chemical pesticides, which has led to the development of resistance to organophosphate and nereistoxin insecticides due to excessive use in the field [7, 13]. Our study provides information and resource to identify and facilitate functional studies of genes responsible for neurotransmitter biosynthesis, transport and degradation at the molecular level. By *do novo* sequencing of the *C. suppressalis* central nervous system transcriptome, we obtained 54,411 assembled unigenes. Among these unigenes, we have identified 32 unigenes encoding 21 enzymes and 11 transporters putatively involved in neurotransmitter signaling systems by local blast. We further confirmed these unigenes via homology search and phylogenetic tree analysis (Tables 2, 3 and Figs. 5, 6 and 7). For each neurotransmitter, we searched for and identified proteins involved in its biosynthesis, packaging, and recycling/degradation. We revealed the expression profiles of enzymes and transporters in various tissues by RT-PCR and qRT-PCR, which indicated that most enzymes were highly expressed in the central nervous system and all the transporters were specifically expressed in the central nervous system. In addition, the transcript abundances of enzymes and transporters in the central nervous system were validated by qRT-PCR (Fig. 8). The high expression levels may reflect their important roles in the central nervous system. Interestingly, the expression of enzymes and transporters also could be detected in hemocytes, these results were consistent with the previous studies that the neurotransmitters may be important molecules bridging the nervous system and immune system [3, 37, 44, 51]. In this study, we summarized how the neurotransmitters are produced, released and recycled between presynaptic terminal and synaptic cleft, and how the enzymes and transporters function in the neurotransmitter signaling systems (Fig. 14). To our knowledge, this is the first study to characterize neurotransmitter signaling pathways comprehensively in rice pest. It is known that the enzymes are prime targets of pesticides because of their important roles in biosynthesis and degradation of the neurotransmitters. In addition, due to their crucial roles in insect nutrition and phylogenetic specificity, neurotransmitter transporters are considered to be excellent targets for the development of lineage-specific and environmentally safe insecticides [89]. Therefore, our study provides a valuable resource of molecular information for future investigations of the functions of neurotransmitter-related genes and developing new potential pesticides for insect pest control.

## Methods

### Insect rearing

The *C. suppressalis* colony has been reared in our laboratory continuously, of which larvae were originally collected from the rice field in Fuyang, Zhejiang Province, China, in 2012. The larvae were reared on artificial diet [90] and kept at 25 ± 1 °C with approximately 80 % relative humidity under a 14:10 light:dark cycle.

### Sample collection and RNA isolation

The fifth instar larvae of *C. suppressalis* were surface-sterilized with 75 % ethanol. Then, the central nervous system (brain, suboesophageal ganglion, thoracic ganglion and abdominal ganglion) samples were individually dissected under saline solution with RNase inhibitor (TaKaRa, Japan) from 100 larvae. Total RNA samples were extracted using TRIzol Reagent (Invitrogen, Carlsbad, CA, USA) following the manufacturer’s instructions and stored in −80 °C. RNA concentration was measured using Qubit® RNA Assay Kit in Qubit® 2.0 Flurometer (Life Technologies, CA, USA). RNA integrity was assessed using the RNA Nano 6000 Assay Kit of the Bioanalyzer 2100 system (Agilent Technologies, CA, USA).

### cDNA library construction

A total amount of 3 μg RNA per sample was used for construction of cDNA library. All samples had RIN (RNA Integrity Number) values above 8. Sequencing of libraries was performed using Illumina TruSeq™ RNA Sample Preparation Kit (Illumia, San Diego, USA) following manufacturer’s recommendations and four index codes were added to attribute sequences to each sample. Briefly, mRNA was purified from total RNA using poly-T oligo-attached magnetic beads. Fragmentation was carried out using divalent cations under elevated temperature in Illumina proprietary fragmentation buffer. First strand cDNA was synthesized using random oligonucleotides and SuperScript II. Second strand cDNA synthesis was subsequently performed using DNA polymerase I and RNase H. Remaining overhangs were converted into blunt ends via exonuclease/polymerase activities and enzymes were removed. After adenylation of 3’ ends of DNA fragments, Illumina PE adapter oligonucleotides were ligated for hybridization. In order to select cDNA fragments of preferentially 200 bp in length, the library fragments were purified with AMPure XP system (Beckman Coulter, Beverly, USA). DNA fragments with ligated adaptor molecules on both ends were selectively enriched using Illumina PCR Primer Cocktail in a 10 cycle PCR reaction. Products were purified (AMPure XP system) and quantified using the Agilent high sensitivity DNA assay on the Agilent Bioanalyzer 2100 system (Agilent Technologies, CA, USA) [91].

### Illumina sequencing, assembly, and annotation

Transcriptome sequencing was carried out on an Illumina HiSeq 2000 platform that generated about 100 bp paired-end (PE100) raw reads (Novogene Bioinformatics Technology Co.Ltd). Raw data were deposited to NCBI Short Read Archive (SRA) database (http://www.ncbi.nlm.nih.gov/Traces/sra/). After removing adaptor sequences, ambiguous ‘N’ nucleotides (with the ratio of ‘N’ to be more than 10 %) and low quality sequences (with quality score to be less than 5), the remaining clean reads were assembled using Trinity software as described for *de novo* transcriptome assembly without reference genome. For homology annotation, non-redundant sequences were subjected to public databases, including NCBI (http://www.ncbi.nlm.nih.gov/) non-redundant protein (Nr) and non-redundant nucleotide (Nt), SwissProt (http://www.ebi.ac.uk/uniprot/), Gene Ontology (GO) (http://www.geneontology.org/), Clusters of Orthologous Groups (COG) (http://www.ncbi.nlm.nih.gov/COG/), and Kyoto Encyclopedia of Genes and Genomes (KEGG) (http://www.genome.jp/kegg/). If results of different databases were conflicted, a priority order of alignments from Nr, Nt, KEGG, SwissProt, GO and COG databases was followed. Comparing to Nr, Nt and Swiss-Prot databases were carried out using BLASTX algorithm with an E-value cut-off of 10^−10^_._ GO terms at 2^nd^ level was used to perform GO annotation. COG and KEGG classifications were done using BLASTX with an E-value cut off of 10^−5^_._

### Identification of putative genes associated with neurotransmitter biosynthesis and transport

We used amino acid sequences of enzymes and transporters from the fruit fly *D. melanogaster* and other invertebrates that are associated with neurotransmitter biosynthesis and transport as queries for BLAST analysis (TBLASTN) to search the candidate sequences of enzymes and transporters in *C. suppressalis*. The BLAST + 2.2.23 software (downloadable from the National Center for Biotechnology Information, Bethesda, MD, USA; ftp://ftp.ncbi.nlm.nih.gov/blast/executables/blast+/) was used for local BLAST to search the assembled unigenes. After the identification of genes, we performed BLASTX and BLASTN programs against non-redundant protein (Nr) and nucleotide sequence (Nt) databases at NCBI to find the homologous sequences in other insects [92].

### Phylogenetic analysis and sequence alignment

To identify potential orthologs of the genes associated with neurotransmitter biosynthesis and transport, we constructed the phylogenetic trees of these putative genes in *C. suppressalis* and other insects. The sequences were aligned using ClustalW2 (http://www.ebi.ac.uk/ Tools/msa/clustalw2/). The tree was drawn using MEGA 5.0 with the maximum likelihood method [93] and the branch support values are expressed as percentages. Multiple sequence alignments of the complete amino acid sequences were performed with ClustalX2 [94] and edited with software GeneDoc. The accession numbers of sequences used in this study were shown in Additional file 11.

### RT-PCR and qRT-PCR

The total RNA were isolated from fifth instar larval central nervous system, gut (foregut, midgut, hindgut, and Malpighian tube), hemocytes and fat body. cDNA was synthesized from 1 μg RNA using TransScript One-Step gDNA Removal and cDNA Synthesis SuperMix (Transgen, Beijing, China) for RT-PCR and qRT-PCR. Specific primers for RT-PCR and qRT-PCR analysis were designed with Primer 3 (http://bioinfo.ut.ee/primer3-0.4.0/) (Additional files 12 and 13). RT-PCR was carried out in a 50 μl reaction containing 5 μl 10 × TaKaRa Ex Taq, 4 μl dNTP Mixture, 0.5 μl TaKaRa Ex Taq (TaKaRa, Japan), 2 μl each primer (10 μM), 1 μl cDNA template, 35.5 μl sterile H_2_O. The PCR cycling profile was: 94 °C for 3 min, followed by 40 cycles of 94 °C for 30 sec, 60 °C for 30 sec, 72 °C for 1 min and a final extension for 10 min at 72 °C. PCR products were separated in 1.5 % agarose gels and stained with ethidium bromide. qRT-PCR was conducted using the CFX Connect™ Real-Time Detection System (Bio-rad, USA). The reference gene, elongation factor 1 alpha (EF-1), was used for normalizing expression of the target gene. qRT-PCR was done in a 25 μl reaction containing 12.5 μl SYBR® Premix Ex Taq™ II (Tli RNaseH Plus) (TaKaRa, Japan), 1 μl each primer (10 μM), 5 μl cDNA template, 5.5 μl sterile H_2_O. The qRT-PCR procedure was 95 °C for 30 sec, followed by 40 cycles of 95 °C for 5 sec and 60 °C for 30 sec. Then, the PCR products were heated to 95 °C for 15 sec, cooled to 60 °C for 1 min and heated to 95 °C for 30 sec and cooled to 60 °C for 15 sec to measure the dissociation curves. Three biological samples of each tissue were used to ensure the reliability and reproducibility.

### qRT-PCR data analysis

The relative quantification in each tissue was calculated using the comparative 2^-ΔΔCT^ method [95]. All data were normalized to endogenous elongation factor 1 alpha level from the same individual samples. In the analysis of the relative expression level in different tissues, the lowest expression level was taken as the calibrator. Thus, the relative expression level in different tissues was assessed by comparing the expression level of each target gene in other tissues to that in the lowest part. The results are presented as the mean of the expression level in three biological replicates. The data of relative expression levels in various tissues were analyzed using one-way analysis of variance (ANOVA), followed by a Tukey’s honestly significant difference (HSD) test when significant differences were tested. All statistical analysis was performed by Data Processing System (DPS) package (Version 9.5) [96].

### Availability of supporting data

The sequences of the unigenes used in the study have been submitted to the NCBI. The transcriptomic data of *C. suppressalis* central nervous system has been submitted to Sequence Read Archive (SRA) database (http://www.ncbi.nlm.nih.gov/sra), and the accession number is SRR2015503.

## Additional files

Additional file 1:
**Summary of distribution of assembled length in** ***C. suppressalis*** **central nervous system transcriptome.**
Additional file 2:
**The identified unigenes with putative roles in** ***C. suppressalis*** **neurotransmitters biosynthesis and transport (Table** 2 **,** 3 **).**
Additional file 3:
**Gene ontology classification of the** ***C. suppressalis*** **central nervous system transcriptome.**
Additional file 4:
**COG functional classification of the** ***C. suppressalis*** **central nervous system transcriptome.**
Additional file 5:
**KEGG functional classification of the** ***C. suppressalis*** **central nervous system transcriptome.** A, Cellular Processes; B, Environmental Information Processing; C, Genetic Information Processing; D, Metabolism; E, Organismal Systems. Additional file 6:
**Amino acid sequence alignment of tyrosine hydroxylase homologues.** The sequences are from CsTH-L (KP657623), CsTH-S (KP657624), MsTH-L (BAF32573.1), MsTH-S (BAF32574.1), DmTH-L (NP_476898.1) and DmTH-S (NP_476897.1). DmTH (Ser32) is phosphorylated by cAMP-dependent protein kinase and is conserved in CsTH (Ser31) (red asterisk). The putative catalytic domain is indicated between two red dotted lines. Alternatively spliced domains are marked with red arrows. Additional file 7:
**Amino acid sequence alignment of acetylcholinesterase homologues.** The sequences are from DmAChE (P07140.1), CsAChE1 (KP657634), BmAChE1 (ABY50088.1), TcAChE1 (ADU33189.1), CsAChE2 (KP657635), BmAChE2 (ABY50089.1), and TcAChE2 (ADU33190.1). The number 1, 2, 3 on the amino acids show the residues forming intramolecular disulfide bonds. The active site triad residues are marked with red filled diamonds. Purple filled triangles indicate the oxyanion hole-forming residues. Black filled circles represent the acylpocket, while open green diamonds mark the peripheral anionic site. The choline binding site is indicated by the blue arrows. The cholinesterase signature sequence is underlined. Additional file 8:
**Amino acid sequence alignment of glutamine synthetase homologues.** The sequences are from CsGS1 (KP657637), BmGS1 (XP_004930366.1), DmGS1 (NP_476570.1), CsGS2 (KP657638), BmGS2 (XP_004929856.1), and DmGS2 (NP_511123.2). Residues involved in binding of glutamate (red filled triangles), ATP (purple filled diamonds), and ammonia (black filled circles) are highlighted. Additional file 9:
**Amino acid sequence alignment of excitatory amino acid transporter homologues.** The sequences are from CsEAAT1 (KP657650), BmEAAT1 (NP_001240824.1), DmEAAT1 (NP_477428.1), CsEAAT2 (KP657651), BmEAAT2 (NP_001240825.1), DmEAAT2 (NP_001162844.1). Additional file 10:
**Amino acid sequence alignment of glutamate decarboxylase homologues.** The sequences are from CsGAD1 (KP657640), BmGAD1 (XP_004925034.1), DmGAD1 (NP_523914.2), CsGAD2 (KP657641), BmGAD2 (XP_004932908.1), and DmGAD2 (NP_001285910.1). Domain “a” is the proposed substrate binding domain. Domain ‘b’ is the ‘decarboxylation’ domain and contains the pyridoxal binding site ‘NPHK’(underlined). The conserved active site residues are marked with purple arrows. The functional residues are indicated by red filled triangles. Additional file 11:
**The accession number of the sequences used in this study.**
Additional file 12:
**Primers used for RT-PCR analysis of enzyme and transporter genes in** ***C. suppressalis*** **.**
Additional file 13:
**Primers used for qRT-PCR analysis of expression levels of enzyme and transporter genes in** ***C. suppressalis*** **.**

## Abbreviations

DA: dopamine
OA: octopamine
TA: tyramine
5HT: serotonin
HA: histamine
MA: monoamine
MAT: monoamine transporter
DAT: dopamine transporter
OAT: octopamine transporter
SERT: serotonin transporter
VMAT: vesicular monoamine transporter
DAR: dopamine receptor
OAR: octopamine receptor
TAR: tyramine receptor
5HTR: serotonin receptor
HAR: histamine receptor
TH: tyrosine hydroxylase
DDC: dopa decarboxylase
TDC: tyrosine decarboxylase
TβH: tyramine beta hydroxylase
HDC: histidine decarboxylase
TRH: tryptophan hydroxylase
TPH: phenylalanine hydroxylase
ebony: NBAD synthetase
tan: NBAD hydrolase
aaNAT: arylalkylamine-N-acetyl transferase
ACh: acetylcholine
Ch: choline
ChT: choline transporter
VAChT: vesicular acetylcholine transporter
nAChR: nicotinic acetylcholine receptor
mAChR: muscarinic acetylcholine receptor
AChE: acetylcholinesterase
ChAT: choline acetyltransferase
Glu: glutamate
Gln: glutamine
EAAT: excitatory amino acid transporter
VGluT: vesicular glutamate transporter
GluR: glutamate receptor
GDH: glutamate dehydrogenase
GLS: glutaminase
GS: glutamine synthetase
GABA: γ-aminobutyric acid
GAT: GABA transporter
VGAT: vesicular GABA transporter
GABAR: GABA receptor
GAD: glutamic acid decarboxylase
GABAT: GABA transaminase
SSADH: succinic semialdehyde dehydrogenas
AC: adenylyl cyclase
DAG: diacylglycerol
IP3: 1,4,5-trisphosphate
PKA: protein kinase A
PKC: protein kinase C
PLC: phospholipase C
ATP: adenosine triphosphate
cAMP: cyclic adenosine monophosphate
