# Supplementary material for: De novo assembly and characterization of central nervous system transcriptome reveals neurotransmitter signaling systems in the rice striped stem borer, Chilo suppressalis
Source: BMC Genomics. 2015 Jul 15;16(1):525. doi: 10.1186/s12864-015-1742-7 (PMC4501067; doi:10.1186/s12864-015-1742-7)
Supplement: Additional file 1: — Summary of distribution of assembled length in C. suppressalis central nervous system transcriptome. [file 12864_2015_1742_MOESM1_ESM.docx]

**Additional file 1: Summary of distribution of assembled length in *C. suppressalis* central nervous system transcriptome.**

|  | **Transcripts** | **Unigenes** |
| --- | --- | --- |
| Min length | 201 | 201 |
| Mean length | 1,330 | 893 |
| Median length | 659 | 410 |
| Max length | 21,491 | 21,491 |
| N50 | 2,647 | 1,808 |
| N90 | 512 | 314 |
| Total Nucleotides | 140,671,344 | 48,589,268 |
