# Supplementary figures and images for: De novo assembly and characterization of central nervous system transcriptome reveals neurotransmitter signaling systems in the rice striped stem borer, Chilo suppressalis
Source: BMC Genomics. 2015 Jul 15;16(1):525. doi: 10.1186/s12864-015-1742-7 (PMC4501067; doi:10.1186/s12864-015-1742-7)

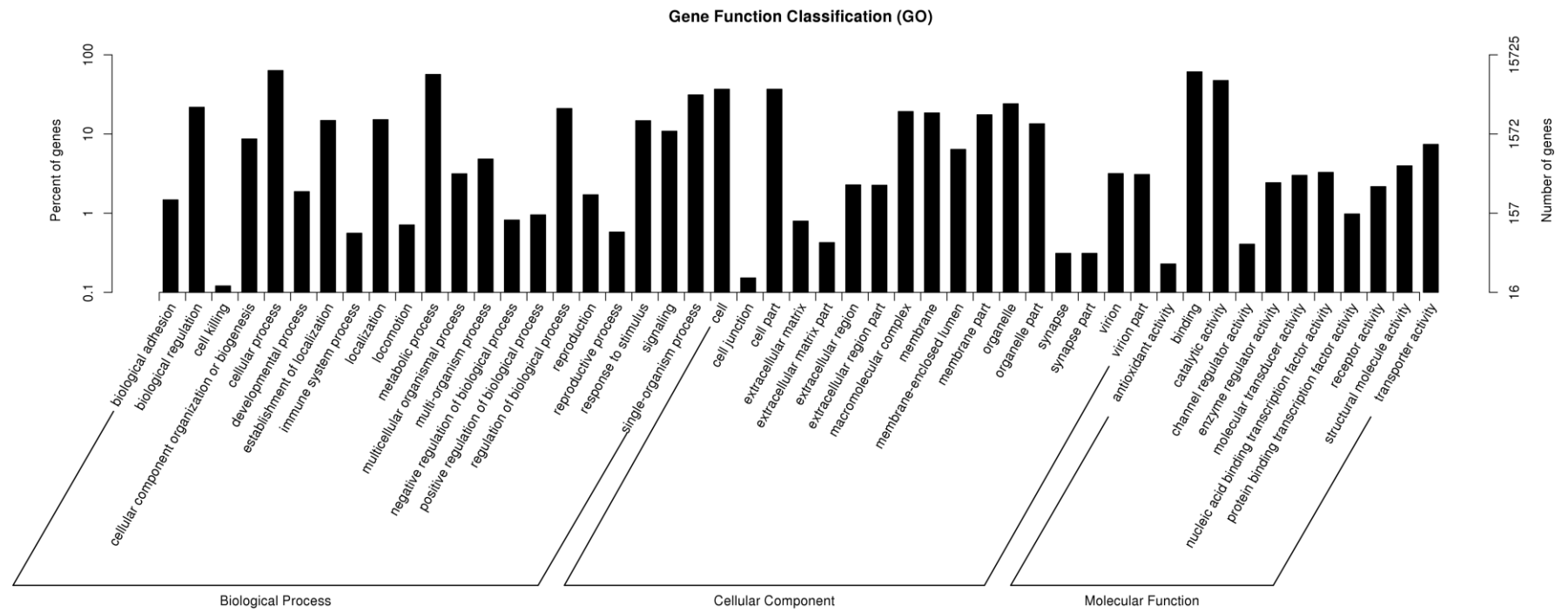

Supplement: Additional file 3: — Gene ontology classification of the C. suppressalis central nervous system transcriptome. [file 12864_2015_1742_MOESM3_ESM.pdf]

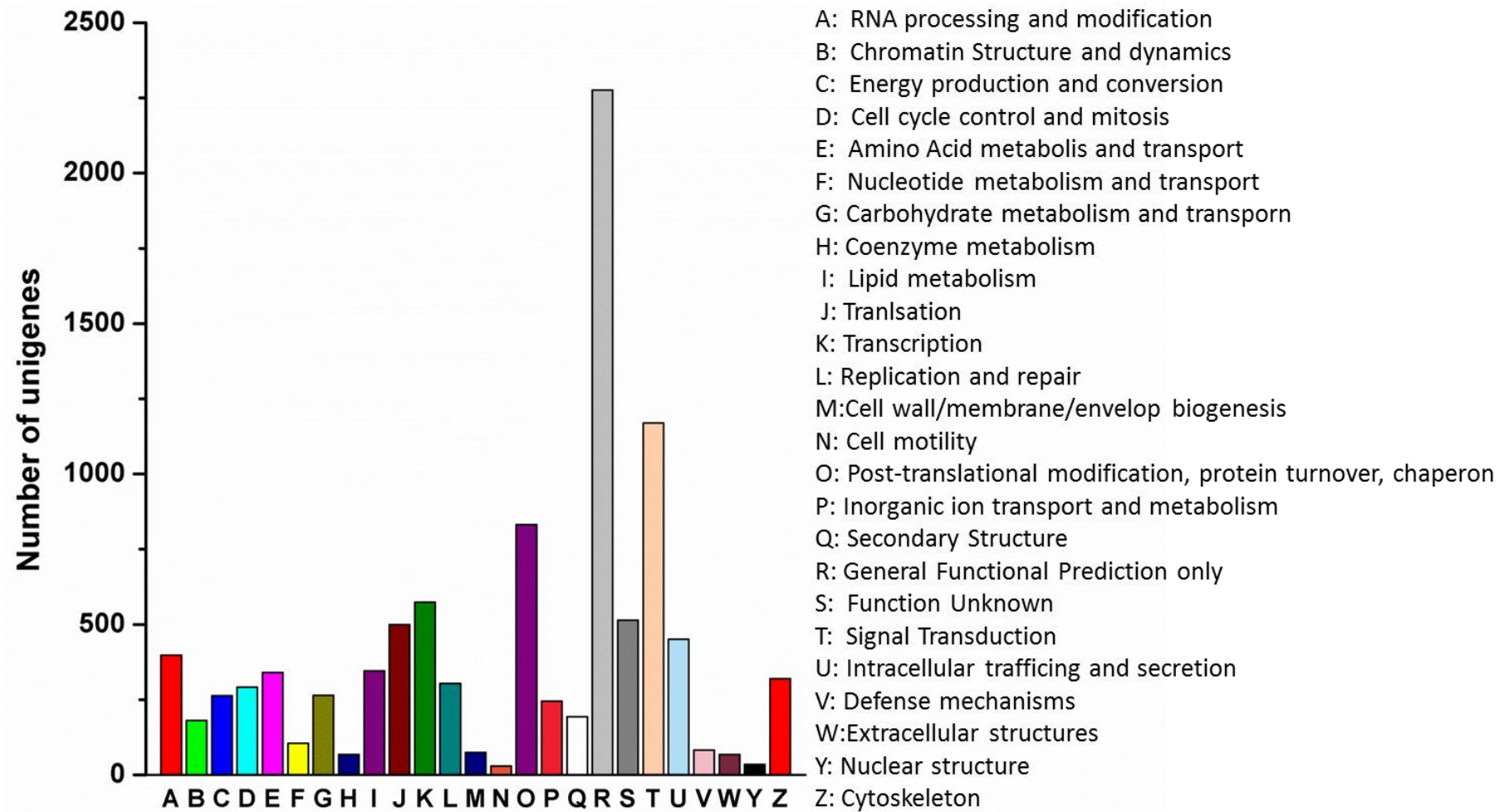

Supplement: Additional file 4: — COG functional classification of the C. suppressalis central nervous system transcriptome. [file 12864_2015_1742_MOESM4_ESM.pdf]

## KEGG Classification

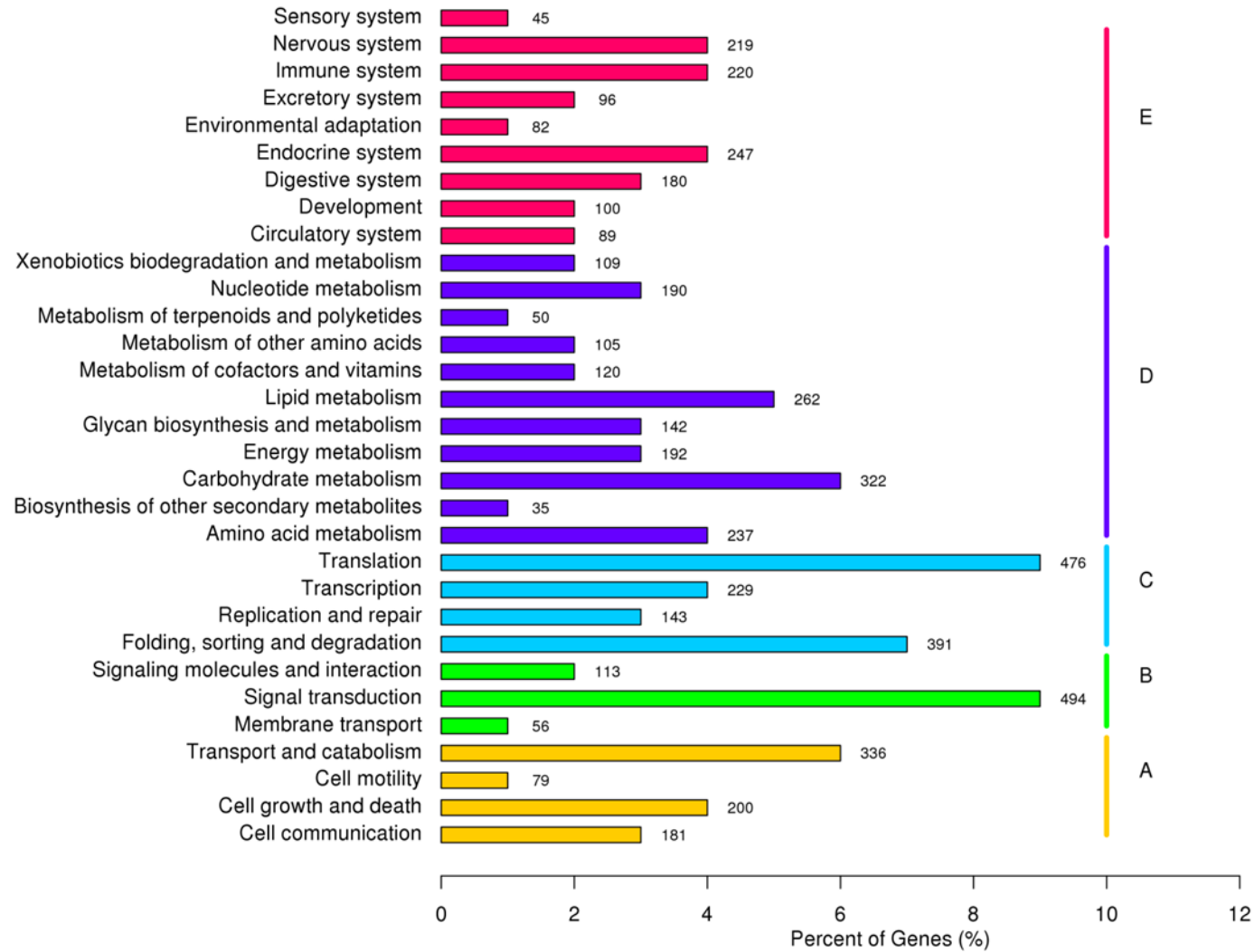

Supplement: Additional file 5: — KEGG functional classification of the C. suppressalis central nervous system transcriptome. A, Cellular Processes; B, Environmental Information Processing; C, Genetic Information Processing; D, Metabolism; E, Organismal Systems. [file 12864_2015_1742_MOESM5_ESM.pdf]
