# Supplementary material for: De novo assembly and characterization of central nervous system transcriptome reveals neurotransmitter signaling systems in the rice striped stem borer, Chilo suppressalis
Source: BMC Genomics. 2015 Jul 15;16(1):525. doi: 10.1186/s12864-015-1742-7 (PMC4501067; doi:10.1186/s12864-015-1742-7)
Supplement: Additional file 6: — Amino acid sequence alignment of tyrosine hydroxylase homologues. The sequences are from CsTH-L (KP657623), CsTH-S (KP657624), MsTH-L (BAF32573.1), MsTH-S (BAF32574.1), DmTH-L (NP_476898.1) and DmTH-S (NP_476897.1). DmTH (Ser32) is phosphorylated by cAMP-dependent protein kinase and is conserved in CsTH (Ser31) (red asterisk). The putative catalytic domain is indicated between two red dotted lines. Alternatively spliced domains are marked with red arrows. [file 12864_2015_1742_MOESM6_ESM.pdf]

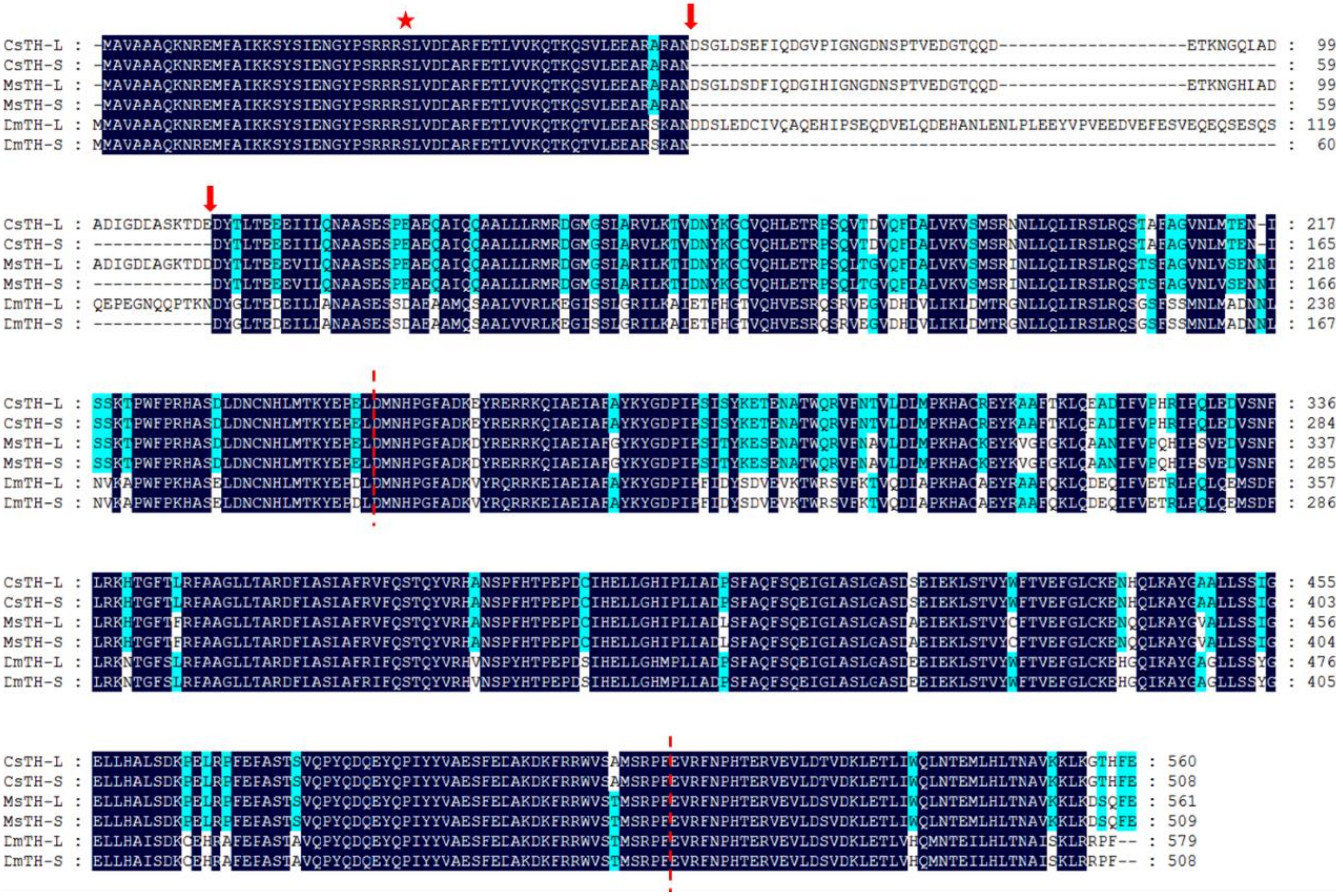
CsTH-L
CsTH-S
MsTH-L
MsTH-S
DmTH-L
DmTH-S
