# Supplementary material for: De novo assembly and characterization of central nervous system transcriptome reveals neurotransmitter signaling systems in the rice striped stem borer, Chilo suppressalis
Source: BMC Genomics. 2015 Jul 15;16(1):525. doi: 10.1186/s12864-015-1742-7 (PMC4501067; doi:10.1186/s12864-015-1742-7)
Supplement: Additional file 7: — Amino acid sequence alignment of acetylcholinesterase homologues. The sequences are from DmAChE (P07140.1), CsAChE1 (KP657634), BmAChE1 (ABY50088.1), TcAChE1 (ADU33189.1), CsAChE2 (KP657635), BmAChE2 (ABY50089.1), and TcAChE2 (ADU33190.1). The number 1, 2, 3 on the amino acids show the residues forming intramolecular disulfide bonds. The active site triad residues are marked with red filled diamonds. Purple filled triangles indicate the oxyanion hole-forming residues. Black filled circles represent the acylpocket, while open green diamonds mark the peripheral anionic site. The choline binding site is indicated by the blue arrows. The cholinesterase signature sequence is underlined. [file 12864_2015_1742_MOESM7_ESM.pdf]

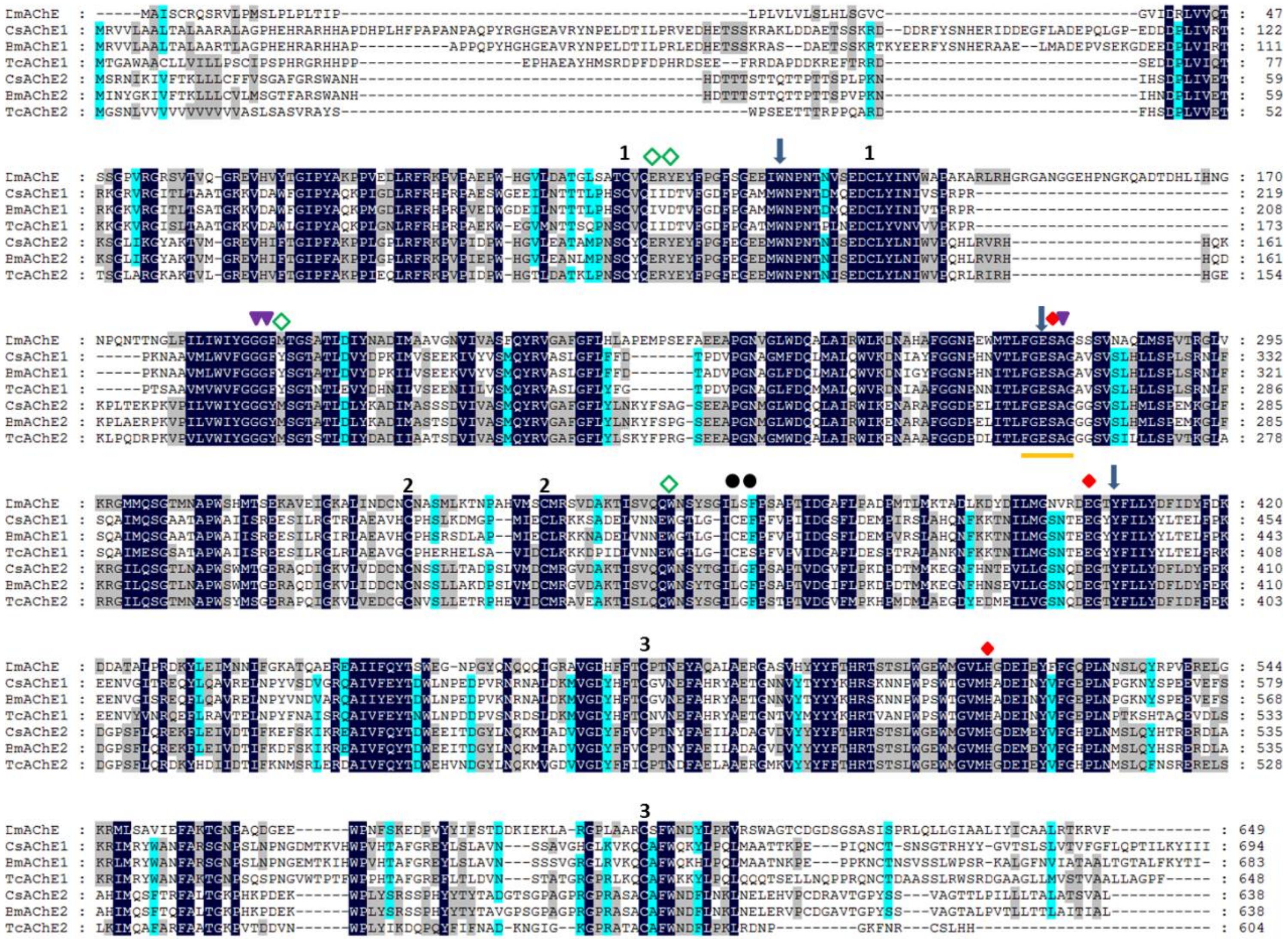
LmAChE
CsAChE1
BmAChE1
TcAChE1
CsAChE2
BmAChE2
TcAChE2
