# Supplementary material for: De novo assembly and characterization of central nervous system transcriptome reveals neurotransmitter signaling systems in the rice striped stem borer, Chilo suppressalis
Source: BMC Genomics. 2015 Jul 15;16(1):525. doi: 10.1186/s12864-015-1742-7 (PMC4501067; doi:10.1186/s12864-015-1742-7)
Supplement: Additional file 8: — Amino acid sequence alignment of glutamine synthetase homologues. The sequences are from CsGS1 (KP657637), BmGS1 (XP_004930366.1), DmGS1 (NP_476570.1), CsGS2 (KP657638), BmGS2 (XP_004929856.1), and DmGS2 (NP_511123.2). Residues involved in binding of glutamate (red filled triangles), ATP (purple filled diamonds), and ammonia (black filled circles) are highlighted. [file 12864_2015_1742_MOESM8_ESM.pdf]

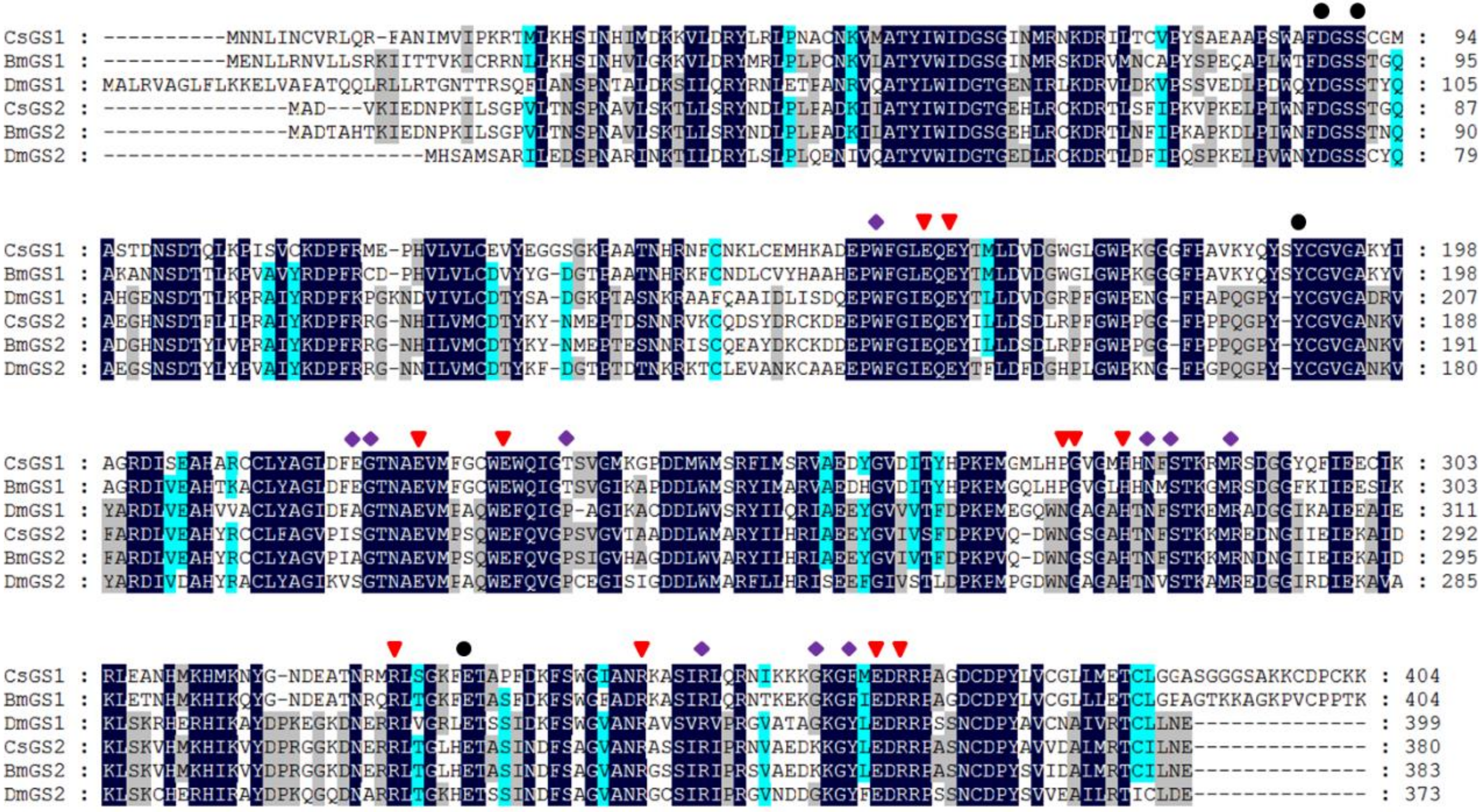
CsGS1 : ----------MNNLINCVRLQR-FANIMVIPKRTMLKHSINHIMDKKVLDRYLRLPNACNKVMATYIWIDGSGINMRNKDRILTCVPYSAEAAPSWAFDGSSCGM : 94
BmGS1 : ----------MENLLRNVLLSRKIITTVKICRRNLLKHSINHVLGKKVLDRYMRLPLPCNKVLATYVWIDGSGINMRSKDRVMNCAPYSPEQAPLWTFDGSSTGQ : 95
DmGS1 : MALRVAGLFLKKELVAFATQQLRLLRTGNTTRSQFLANSPNTALDKSILQRYRNLETFANRVQATYLWIDGTGENIRLKDRVLDKVPSSVEDLPDWQYDGSSTYQ : 105
CsGS2 : ---------------MAD---VKIEDNPKILSGPVLTNSPNAVLSKTLLSRYNDLPLFADKIIATYIWIDGTGEHLRCKDRTLSFIPKVPKELPIWNFDGSSTGQ : 87
BmGS2 : ---------------MADTAHTKIEDNPKILSGPVLTNSPNAVLSKTLLSRYNDLPLFADKIIATYIWIDGSGEHLRCKDRTLNFIPKAPKDLPIWNFDGSSTNQ : 90
DmGS2 : -------------------------MHSAMSARILEDSPNARINKTIILDRYLSLPLQENIVQATYVWIDGTGEDLRCKDRTLDFIPQSPKELPVWNYDGSSCYQ : 79
CsGS1 : ASTDNSDTQLKPISVCKDPFRME-PHVLVLCEVYEGGSGKPAATNHRNFCNKLCEMHKADEPWFGLEQEYTMLDVDGWGLGWPKGGGFPAVKYQYSYCGVGAKYI : 198
BmGS1 : AKANNSDTTLKPVAVYRDPFRCD-PHVLVLCDVYYG-DGTPAATNHRKFCNDLCVYHAAHEPWFGLEQEYTMLDVDGWGLGWPKGGGFPAVKYQYSYCGVGAKYV : 198
DmGS1 : AHGENSDTTLKPRAIYRDPFKPGKNDVIVLCDTYSA-DGKPTASNKRAAFCAAIDLISDQEPWFGIEQEYTLLDVDGRPFGWPENG-FPAPQGPY-YCGVGADRV : 207
CsGS2 : AEGHNSDTFLIPRAIYKDPFRRG-NHILVMCDTYKY-NMEPTDSNNRVKCQDSYDRCKDEEPWFGIEQEYILLDSDLRPFGWPPGG-FPPPQGPY-YCGVGANKV : 188
BmGS2 : ADGHNSDTYLVPRAIYKDPFRRG-NHILVMCDTYKY-NMEPTESNNRISCQEAYDKCKDDEPWFGIEQEYILLDSDLRPFGWPPGG-FPPPQGPY-YCGVGANKV : 191
DmGS2 : AEGSNSDTYLYPVAIYKDPFRRG-NNILVMCDTYKF-DGTPTDTNKRKTCLEVANKCAAEEPWFGIEQEYTFLDFDGHPLGWPKNG-FPGPQGPY-YCGVGANKV : 180
CsGS1 : AGRDISEAHARCCLYAGLDFEGTNAEVMFGCWEWQIGTSVGMKGPDLMWMSRFIMSRVAEDYGVDITYHPKPMGMLHPGVGMHHNFSTKRMRSDGGYQFIEECIK : 303
BmGS1 : AGRDIVEAHTKACLYAGLDFEGTNAEVMFGCWEWQIGTSVGIKAPDDLWMSRYIMARVAEDHGVDITYHPKPMGQLHPGVGLHHNMSTKGMRSDGGFKIIEESIK : 303
DmGS1 : YARDIVEAHVVACLYAGIDFAGTNAEVMPAQWEFQIGP-AGIKACDDLWVSRYILQRIAEEYGVVVTFDPKPMEGQWNGAGAHTNFSTKEMRADGGIKAIEEAIE : 311
CsGS2 : FARDIVEAHYRCCLFAGVPISGTNAEVMPSQWEFQVGPSVGVTAADDLWMARYILHRIAEEYGVIVSFDPKPVQ-DWNGSGAHTNFSTKKMREDNGIIEIEKAID : 292
BmGS2 : FARDIVEAHYRCCLYAGVPIAGTNAEVMPSQWEFQVGPSIGVHAGDDLWVARYILHRIAEEYGVIVTFDPKPVQ-DWNGSGAHTNFSTKKMRNDNGIIEIEKAID : 295
DmGS2 : YARDIVDAHYRACLYAGIKVSGTNAEVMPAQWEFQVGPCEGISIGDDLWMARFLLHRISEEFGIVSTLDPKPMPGDWNGAGAHTNVSTKAMREDGGIRDIEKAVA : 285
CsGS1 : RLEANHMKHMKNYG-NDEATNRMRLSGKFETAPFDKFSWGIANRKASIRLQRNIKKKGKGFMEDRRPAGDCDPYLVCGLIMETCLGGASGGGSAKKCDPCKK : 404
BmGS1 : KLETNHMKHIKQYG-NDEATNRQRLTGKFETASFDKFSWGFADRKASIRLQRNTKEKGKGFIEDRRPAGDCDPYLVCGLLIETCLGFAGTKKAGKPVCPPTK : 404
DmGS1 : KLSKRHERHIKAYDPKEGKDNERRLVGRLETSSIDKFSWGVANRAVSVRVPRGVATAGKGYLEDRRPSSNCDPYAVCNAIVRTCLLNE-------------- : 399
CsGS2 : KLSKVHMKHIKVYDPRGGKDNERRLTGLHETASINDFSAGVANRASSIRIPRNVAEDKKGYLEDRRPASNCDPYAVVDAIMRTCILNE-------------- : 380
BmGS2 : KLSKVHMKHIKVYDPRGGKDNERRLTGLHETASINDFSAGVANRGSSIRIPRSVAEDKKGYLEDRRPASNCDPYSVIDAIMRTCILNE-------------- : 383
DmGS2 : KLSKCHERHIRAYDPKQGQDNARRLTGKHETSSINDFSAGVANRGCSIRIPRGVNDDGKGYFEDRRPSSNCDPYSVVEAILRTICLDE-------------- : 373
