# Supplementary material for: De novo assembly and characterization of central nervous system transcriptome reveals neurotransmitter signaling systems in the rice striped stem borer, Chilo suppressalis
Source: BMC Genomics. 2015 Jul 15;16(1):525. doi: 10.1186/s12864-015-1742-7 (PMC4501067; doi:10.1186/s12864-015-1742-7)
Supplement: Additional file 9: — Amino acid sequence alignment of excitatory amino acid transporter homologues. The sequences are from CsEAAT1 (KP657650), BmEAAT1 (NP_001240824.1), DmEAAT1 (NP_477428.1), CsEAAT2 (KP657651), BmEAAT2 (NP_001240825.1), DmEAAT2 (NP_001162844.1). [file 12864_2015_1742_MOESM9_ESM.pdf]

```
CsEAAT1 : ----------------MPLQLPRNRCAVFMRENLLTLMTVIGVLSGTLLGWGLRSSGYEWSKREVMYFQFPGELFLRMLKCLIVPLLVSSIVSAIGSLDLSLSGKVGLRAIIYY :  98
BmEAAT1 : ----------------MPLQFNRKACPKFIRENLLTILTVVGVAAGTLLGCGLRASGHQWSKRDVMYFQFPGELFLRMLKSLIVPLLVSSIVSAIGSLDLSLSGKVGLRAIIYY :  98
DmEAAT1 : ---------------MTRPKQDGGKFKAFMQENVLTMATVIGVFVGGLIGFIIKNSTGEWSKREIMYISFPGEIFLRMLKCLIVPLLVSSITSAIGGLDLSMSSKIATRAITYY :  99
CsEAAT2 : MGP---GDGGTKTTEDRMAPFEPTGARKWFIDNIMLVITLVGVIAGIALGFGLR--PYDLGPLALMMISYPGELFMRLLKLMILPLIIASLIAGSASLNAKMSGKIAVRTLLYF : 109
BmEAAT2 : MGP---GDRGPKTTEERSAPLEPTGWRKCVIHNTMLVVTLVGVVAGIALGFGLR--PYQLGPDTLVLISYPGELFMRLLKLMILPLIIASLIAGSASLNAKMSGRIAVRTLVYF : 109
DmEAAT2 : MGPPTSTELPPKTTECSLAPVELTGYRRWLSENLMLLVTLSGVLLGVILGLSLR--PLNLHGDSIMLISYPGELFMRVLKLMILPLVISSLIAGSASLNAKMNGKIALRTLVYF : 112

CsEAAT1 : MTTTVCAVMLGIALVTTIKPGKDPEAYQQPQPNATKLITKETLTSDTLLDLIRNVFPENIAQATIASYRTKLSYDEKDPTHIKGQLETYKINS--------------------D : 192
BmEAAT1 : MTTTVCAVMLGIALVTTIKPGSNSKGNEE--LNTTKIVTKDTLTSDTLLDLLRNVFPENIPQATIASYRTKLIYDPKDPKMIKGQLETYKIDG--------------------G : 190
DmEAAT1 : FVTTISAVILGICLVTTLRPGQGAKIVET--QTESIDKASKVLTPDTLMDLVRNMFTDNIIQSTMFQHRTEIYEN--TSISFAQPMENWEFKS--------------------A : 189
CsEAAT2 : ILTSMFNASLGILVATLIHPGKPD---LRDSSIGVTIGRRDHSILDSLLDIGRNIFPDNIVQAAFQQAHTVYAESFALFGK-NASENDTVP----------------ALVRVIS : 203
BmEAAT2 : ILTSMFNAFLGIVMAVLIHPGQPE---LKEDVLVAAGSKKDHSILDSLLDIGRNIFPDNIVQAAFQQAHTVYVEETTLIAK-NSTENGTVP----------------TLVRVVS : 203
DmEAAT2 : ASTSFFNAALGIALVLLIHPGNPD---LHN-ADDRSTDRRAVNLLDSLLDIGRNVFPDNIFQASIQQAHTVYLPKPSILHVFNETMNDTIASGSEAQRLSEDLTEDVVLVRDIQ : 222

CsEAAT1 : YPSGSNVLGLVCFSVVLGITLGKMGEKGRPLQNFFHSFSEAMMIITGWVIWLSPLGVFFLVTAKIMEIDSFSELVGRLGLYFMTVLLGLFLHGFGTLSILFIIATRKLPCRYIA : 306
BmEAAT1 : YPFGSNVLGLVCFSIVLGITLGKMGDLSRPLQQFFHSLSEAMMIITGWVIWMSPLGVFFLVTAKIMEIDSFADLVGRLGKYFLTVLLGLFLHGFGTLSILFIIATKKLPCRYLA : 304
DmEAAT1 : QREGSNVLGLVMFSVILGTTIGRMREKGQLLQDFFTTLSEAMMTITSWVIWISPLGVAFLIAAKIIEMESIAATIQSLGWYFITVMIGLFLHGFGTIAVIFFLGTRRLPYRYIA : 303
CsEAAT2 : YRSGTNTLGLVFFCLVFGSLLGTLGPKGKVVIDFFQAIFEVIMKMVTGVMWLTPVGVSSVIAGKILGLSNVAQVMSQLAWFIATVAVGVEFYQLIVMQLIYFVVLRKNPYKFYW : 317
BmEAAT2 : YRSGTNTLGLVFFCLVFGSLLGSLGAKGQVVIDFFQAIFEVIMKMVAGVMWFTPFGVSSIIAGKILGVADVAHVMSQLAWFIATVAVGVELYQLIVMQLIYFIVVKKNPYKFYW : 317
DmEAAT2 : YRSGTNTLGIVFFCLVFGTFLGTIGQKGQVVVDFFAAIFEVIMKMVTCVMWLTPVGISSVIAGKILSVGDLGLVMSQLMWFIVTVAIGVEIYQFVVMCAIYFVVVRRNPFKFYA : 336

CsEAAT1 : KMGQVMATAFGTASSSATMPITIGCCDE-MGLDPRITRFVIPIGATINMDGTALYEAVAAIFIAQLRKVEMSFGKIVAVSVTATAASIGAAGIPQAGLVTMVMVLDTVNLFAED : 419
BmEAAT1 : KMGQVMATAFGTAS-----------------------RFVIPIGATINMDGTALYEAVAAIFIAQMREVEMTFGKIIAVSVTATAASIGAAGIPQAGLVTMVMVLDTVNLFAED : 395
DmEAAT1 : KLSQVIATAFGTGSSSATMPLTIKCLDN-MGIDPRVTRFVIPVGATINMDGTALYEAVAALFIAQYREMSYSFGTIVAVSITATAASIGAAGIPQAGLVTMVMVLDTVGLEPKD : 416
CsEAAT2 : GLSHAMLTASATASTAAALPVTFRAMEGPLRVDPRITRFVLPIGCNINMDGTALFIAVASMFVCQMNLIPLGFPQIATIFLTCTAASVSSASVPSAALVLLLVVIAALAFAHD : 431
BmEAAT2 : GLSHAMLTASATASTAAALPVTFKAMEGPLRIDVRITRFVLPIGCNINMDGTALFIAIAAVFICQMNNMTLGFAQIATIFLTSTAASLSSASVPSAALVLLLVVLSSVLAFAQD : 431
DmEAAT2 : GLIQAMLTAFATASTAAALPITFRCMDEKLKVDPRITRFVLPIGCNINMDGTALYIAVASIFIAQMSGMVLGFGELLTVLLTSTAASMSSASVPSAALVLLLVVLTAILAPVQD : 450

CsEAAT1 : VSIILAVDWLLDRFRTTINVVCDALGAIIVSSLSQGDIEKNRAEH----------------------------------NERELANTHEITEVEKGEH------------- : 483
BmEAAT1 : VSIILAVDWLLDRFRTTINVVCDALGAIIVTSLSQGDIDKSRAIQ----------------------------------NEREAACAQEITELEKGDL------------- : 459
DmEAAT1 : VSLIIAVDWLLDRFRTTINVMCDALGTILVNHLSKNDIAS---------------------------------------VDRLNAEPHEILELGPNGHEMKE--------- : 479
CsEAAT2 : VSLLFAVDWLVDRIRTTNNMIGDCYAAAVVEHLSKNEIMACDAISN-----------EPGNNGTTPTLNTEVDLGIVTPSRKSIASDDVIIDMHYSGHHCNSIKRI----- : 526
BmEAAT2 : VSLLFAVDWLVDRIRTTNNMIGDCYAAAVVEHLSKNEIMACDAAS------------------------------IVSIRYPQTQPTEFIAGS--SQWVAFPIRW------ : 504
DmEAAT2 : VTLLFAVDWFVDRIRTTNNMIGDCYTAAVVEELSRKELMALDAASVNYQDMFAGTPNGHGHGHHDGGLLEGQTELETSSKCVMTMTDSVVVDISAVMNNVNLQQEHCNRRV : 561
```
