# Supplementary material for: De novo assembly and characterization of central nervous system transcriptome reveals neurotransmitter signaling systems in the rice striped stem borer, Chilo suppressalis
Source: BMC Genomics. 2015 Jul 15;16(1):525. doi: 10.1186/s12864-015-1742-7 (PMC4501067; doi:10.1186/s12864-015-1742-7)
Supplement: Additional file 10: — Amino acid sequence alignment of glutamate decarboxylase homologues. The sequences are from CsGAD1 (KP657640), BmGAD1 (XP_004925034.1), DmGAD1 (NP_523914.2), CsGAD2 (KP657641), BmGAD2 (XP_004932908.1), and DmGAD2 (NP_001285910.1). Domain “a” is the proposed substrate binding domain. Domain ‘b’ is the ‘decarboxylation’ domain and contains the pyridoxal binding site ‘NPHK’(underlined). The conserved active site residues are marked with purple arrows. The functional residues are indicated by red filled triangles. [file 12864_2015_1742_MOESM10_ESM.pdf]

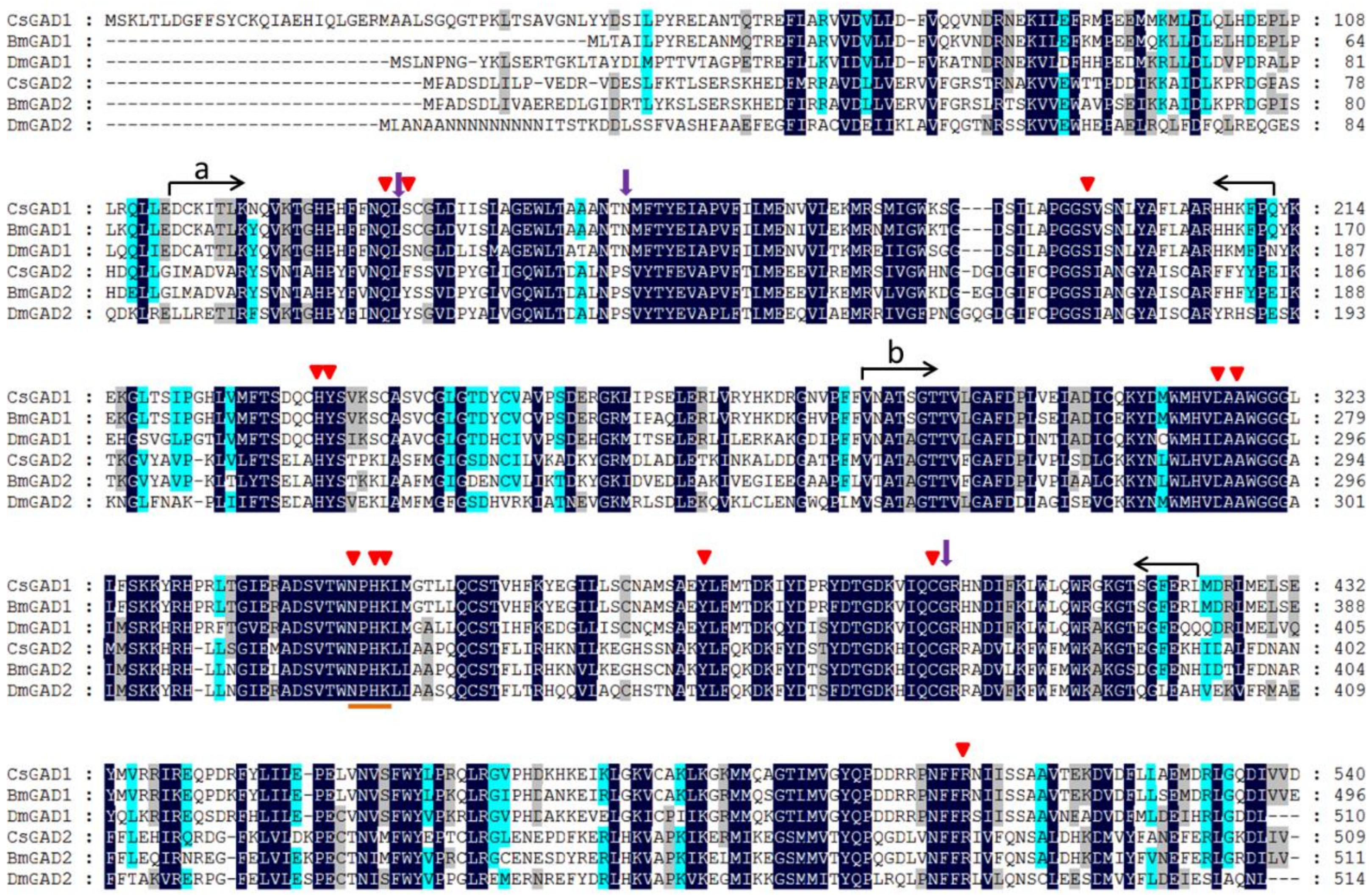

CsGAD1 : MSKLTLDGFFSYCKQIAEHIQLGERMAALSGQGTPKLTSAVGNLYYDSILPYRELANTQTREFIARVVDVLLD-FVQQVNDRNEKILEFRMPEEMMKMLDLQLHDEPLP : 108
BmGAD1 : --MLTAILPYRELANMQTREFIARVVDVLLD-FVQKVNDRNEKILEFKMPEEMQKLLDLELHDEPLP : 64
DmGAD1 : --------------------------MSLNPNG-YKLSERTGKLTAYDLMPTTVTAGPETREFLLKVIDVLLD-FVKATNDRNEKVLDFHHPEDMKRLLDLDVPDRALP : 81
CsGAD2 : -----------------------------MFADSDLILP-VEDR-VDESLFKTLSERSKHEDFMRRAVDLLVERVVFGRSTRNAKVVEWTTPDDIKKAIDLKPRDGFAS : 78
BmGAD2 : -----------------------------MFADSDLIVAEREDLGIDRTLYKSLSERSKHEDFIRRAVDLLVERVVFGRSLRTSKVVEWAVPSEIKKAIDLKPRDGPIS : 80
DmGAD2 : -------------------------MIANAANNNNNNNNNITSTKDDLSSFVASHFAAEFEGFIRACVDEIIKIAVFQGTNRSSKVVEWHEFAELRQLFDFQLREQGES : 84
a
CsGAD1 : LRQLLEDCKITLKNQVKTGHPHFFNQLSCGLDIISIAGEWLTAAANTNMFTYEIAPVFILMENVVLEKMRSMIGWKSG---DSILAPGGSVSNLYAFLAARHHKFPQYK : 214
BmGAD1 : LKQLLEDCKATLKYQVKTGHPHFFNQLSCGLDVISIAGEWLTAAANTNMFTYEIAPVFILMENIVLEKMRNMIGWKTG---DSILAPGGSVSNLYAFLAARHHKFPQYK : 170
DmGAD1 : LQQLIEDCATTLKYQVKTGHPHFFNQLSNGLDLISMAGEWLTATANTNMFTYEIAPVFILMENVVLTKMREIIGWSGG---DSILAPGGSISNLYAFLAARHKMFPNYK : 187
CsGAD2 : HDQLLGIMADVARYSVNTAHPYFVNQLFSSVDPYGLIGQWLTDALNPSVYTFEVAPVFTLMEEEVLREMRSIVGWHNG-DGDGIFCPGGSIANGYAISCARFFYYPEIK : 186
BmGAD2 : HDELLGIMADVARYSVNTAHPYFVNQLYSSVDPYGLVGQWLTDALNPSVYTYEVAPVFTLMEEEVLKEMRVLVGWKDG-EGDGIFCPGGSIANGYAISCARFHFYPEIK : 188
DmGAD2 : QDKLRELLRETIRFSVKTGHPYFINQLYSGVDPYALVGQWLTDALNPSVYTYEVAPLFTLMEEQVIAEMRRIVGFPNGGQGDGIFCPGGSIANGYAISCARYRHSPESK : 193
b
CsGAD1 : EKGLTSIPGHLVMFTSDQCHYSVKSCASVCGLGTDYCVAVPSDERGKLIPSELERLVRYHKDRGNVPFFVNATSGTTVLGAFDPLVEIADICQKYDMWMHVDAAWGGGL : 323
BmGAD1 : EKGLTSIPGHLVMFTSDQCHYSVKSCASVCGLGTDYCVCVPSDERGRMIPAQLERLVRYHKDKGHVPFFVNATSGTTVLGAFDPLSEIADICEKYDMWMHVDAAWGGGL : 279
DmGAD1 : EHGSVGLPGTLVMFTSDQCHYSIKSCAAVCGLGTDHCIVVPSDEHGKMITSELERLILERKAKGDIPFFVNATAGTTVLGAFDDINTIADICQKYNCWMHIDAAWGGGL : 296
CsGAD2 : TKGVYAVP-KLVLFTSELAHYSTPKLASFMGIGSDNCILVKADKYGRMDLADLETKINKALDDGATPFMVTATAGTTVFGAFDPLVPLSDLCKKYNLWLHVDAAWGGGA : 294
BmGAD2 : TKGVYAVP-KLTLYTSELAHYSTKKLAAFMGIGDENCVLIKTDKYGKIDVEDLEAKIVEGIEEGAAPFLVTATAGTTVFGAFDPLVPIAALCKKYNLWLHVDAAWGGGA : 296
DmGAD2 : KNGLFNAK-PLIIFTSELAHYSVEKLAMFMGFGSDHVRKIATNEVGKMRLSDLEKQVKLCLENGWQPIMVSATAGTTVLGAFDDLAGISEVCKKYNMWMHVDAAWGGGA : 301
CsGAD1 : LFSKKYRHPRLTGIERADSVTWNPHKIMGTLLQCSTVHFKYEGILLSCNAMSAEYLFMTDKIYDPRYDTGDKVIQCGRHNDIFKLWLQWRGKGTSGFERLMDRIMELSE : 432
BmGAD1 : LFSKKYRHPRLTGIERADSVTWNPHKIMGTLLQCSTVHFKYEGILLSCNAMSAEYLFMTDKIYDPRFDTGDKVIQCGRHNDIFKLWLQWRGKGTSGFERLMDRIMELSE : 388
DmGAD1 : IMSRKHRHPRFTGVERADSVTWNPHKIMGALLQCSTIHFKEDGLLISCNQMSAEYLFMTDKQYDISYDTGDKVIQCGRHNDIFKLWLQWRAKGTEGFEQQQDRIMELVQ : 405
CsGAD2 : MMSKKHRH-LLSGIEMADSVTWNPHKLIAAPQQCSTFLIRHKNILKEGHSSNAKYLFQKDKFYDSTYDTGDKHIQCGRRADVLKFWFMWKAKGTEGFEKHIDALFDNAN : 402
BmGAD2 : IMSKKHRH-LLNGIELADSVTWNPHKLIAAPQQCSTFLIRHKNVLKEGHSCNAKYLFQKDKFYDTSYDTGDKHIQCGRRADVLKFWFMWKAKGSDGFENHIDTLFDNAR : 404
DmGAD2 : IMSKKYRH-LLNGIERADSVTWNPHKLIAASQQCSTFLTRHQQVIAQCHSTNATYLFQKDKFYDTSFDTGDKHIQCGRRADVFKFWFMWKAKGTQGLEAHVEKVFRMAE : 409
CsGAD1 : YMVRRIREQPDRFYLILE-PELVNVSFWYLPRQLRGVPHDKHKEIKIGKVCAKLKGKMMCAGTIMVGYQPDDRRPNFFRNIISSAAVTEKDVDFLIAEMDRIGQDIVVD : 540
BmGAD1 : YMVRRIKEQPDKFYLILE-PELVNVSFWYLPKQLRGIPHLANKEIRIGKVCAKLKGRMMQSGTIMVGYQPDDRRPNFFRNIISSAAVTEKDVDFLLSEMDRIGQDIVVE : 496
DmGAD1 : YQLKRIREQSDRFHLILE-PECVNVSFWYVPKRLRGVPHLAKKEVELGKICPIIKGRMMQKGTIMVGYQPDDRRPNFFRSIISSAAVNEADVDEMLDEIHRIGDDL--- : 510
CsGAD2 : FFLEHIRQRDG-FKLVLDKPECTNVMFWYEPTCLRGLENEPDFKERLHKVAPKIKERMIKEGSMMVTYQPQGDLVNFFRIVFQNSALDHKLMVYFANEFERLGKDLIV- : 509
BmGAD2 : FFLEQIRNREG-FELVIEKPECTNIMFWYVPRCLRGCENESDYRERLHKVAPKIKELMIKEGSMMVTYQPQGDLVNFFRIVFQNSALDHKLMIYFVNEFERLGRDILV- : 511
DmGAD2 : FFTAKVRERPG-FELVLESPECTNISFWYVPPGLREMERNREFYDRLHKVAPKVKEGMIKKGSMMITYQPLRQLPNFFRLVLQNSCLEESLMVYFLDEIESLAQNL--- : 514
