# Supplementary material for: De novo assembly and characterization of central nervous system transcriptome reveals neurotransmitter signaling systems in the rice striped stem borer, Chilo suppressalis
Source: BMC Genomics. 2015 Jul 15;16(1):525. doi: 10.1186/s12864-015-1742-7 (PMC4501067; doi:10.1186/s12864-015-1742-7)
Supplement: Additional file 11: — The accession number of the sequences used in this study. [file 12864_2015_1742_MOESM11_ESM.docx]

**Additional file 11:** **The accession number of the sequences used in this study.**

| **Gene ID** | **Accession No.** | **Description** |
| --- | --- | --- |
| BmTH | NP_001138794.1 | tyrosine hydroxylase [*Bombyx mori*] |
| TcTH | NP_001092299.1 | tyrosine hydroxylase [*Tribolium castaneum*] |
| DmTH | NP_476898.1 | pale, isoform B [*Drosophila melanogaster*] |
| BmDDC | NP_001037174.1 | aromatic-L-amino-acid decarboxylase [*Bombyx mori*] |
| TcDDC | ABU25222.1 | dopa decarboxylase [*Tribolium castaneum*] |
| DmDDC | AAF53763.1 | dopa decarboxylase, isoform B [*Drosophila melanogaster*] |
| Bmebony | BAH11147.1 | ebony protein [*Bombyx mori*] |
| Tcebony | ACN43327.1 | ebony [*Tribolium castaneum*] |
| Dmebony | ABO27243.1 | ebony [*Drosophila melanogaster*] |
| Bmtan | NP_001170882.1 | tan protein [*Bombyx mori*] |
| Tctan | XP_971848.1 | PREDICTED: uncharacterized protein LOC660531 [*Tribolium castaneum*] |
| Dmtan | NP_572543.1 | tan, isoform A [*Drosophila melanogaster*] |
| BmaaNAT | NP_001073122.1 | arylalkylamine N-acetyltransferase [*Bombyx mori*] |
| TcaaNAT | NP_001139380.1 | dopamine N acetyltransferase isoform 1 [*Tribolium castaneum*] |
| DmaaNAT | NP_523839.2 | dopamine N acetyltransferase, isoform A [*Drosophila melanogaster*] |
| BmTDC | XP_004924879.1 | PREDICTED: aromatic-L-amino-acid decarboxylase-like [*Bombyx mori*] |
| TcTDC | XP_008198031.1 | PREDICTED: aromatic-L-amino-acid decarboxylase-like [*Tribolium castaneum*] |
| DmTDC1 | NP_610226.2 | tyrosine decarboxylase 1 [*Drosophila melanogaster*] |
| DmTDC2 | NP_724489.1 | tyrosine decarboxylase 2 [*Drosophila melanogaster*] |
| BmTβH | NP_001243923.1 | tyramine beta hydroxylase precursor [*Bombyx mori*] |
| TcTβH | XP_974169.1 | PREDICTED: tyramine beta-hydroxylase [*Tribolium castaneum*] |
| DmTβH | AAO41640.1 | tyramine beta hydroxylase, isoform B [*Drosophila melanogaster*] |
| BmTPH | NP_001274766.1 | phenylalanine hydroxylase [*Bombyx mori*] |
| TcTPH | XP_967025.1 | PREDICTED: protein henna [*Tribolium castaneum*] |
| DmTPH | NP_523963.2 | henna, isoform A [*Drosophila melanogaster*] |
| BmTRH | XP_004929955.1 | PREDICTED: tryptophan 5-hydroxylase 1-like [*Bombyx mori*] |
| TcTRH | XP_967413.1 | PREDICTED: tryptophan 5-hydroxylase 1 [*Tribolium castaneum*] |
| DmTRH | NP_612080.1 | tryptophan hydroxylase [*Drosophila melanogaster*] |
| AmHDC | EFN88410.1 | Histidine decarboxylase [*Harpegnathos saltator*] |
| TcHDC | XP_975682.1 | PREDICTED: histidine decarboxylase isoform X1 [*Tribolium castaneum*] |
| DmHDC | NP_001260856.1 | histidine decarboxylase, isoform C [*Drosophila melanogaster*] |
| BmChAT | BAO23491.1 | choline acetyltransferase, partial [*Bombyx mori*] |
| TcChAT | XP_008201281.1 | PREDICTED: choline O-acetyltransferase [*Tribolium castaneum*] |
| DmChAT | NP_996239.2 | choline acetyltransferase, isoform B [*Drosophila melanogaster*] |
| BmAChE1 | ABY50088.1 | acetylcholinesterase type 1 [*Bombyx mori*] |
| TcAChE1 | ADU33189.1 | acetylcholinesterase 1 [*Tribolium castaneum*] |
| BmAChE2 | ABY50089.1 | acetylcholinesterase type 2 [*Bombyx mori*] |
| TcAChE2 | ADU33190.1 | acetylcholinesterase 2 [*Tribolium castaneum*] |
| DmAChE | P07140.1 | acetylcholinesterase [*Drosophila melanogaster*] |
| BmGLS | XP_004930135.1 | PREDICTED: glutaminase kidney isoform, mitochondrial-like isoform X2 [*Bombyx mori*] |
| TcGLS | XP_967231.3 | PREDICTED: glutaminase kidney isoform, mitochondrial isoform X2 [*Tribolium castaneum*] |
| DmGLS | ABO52846.1 | IP17795p, partial [*Drosophila melanogaster*] |
| BmGS1 | XP_004930366.1 | PREDICTED: glutamine synthetase 1, mitochondrial-like [*Bombyx mori*] |
| DmGS1 | NP_476570.1 | glutamine synthetase 1, isoform B [*Drosophila melanogaster*] |
| BmGS2 | XP_004929856.1 | PREDICTED: glutamine synthetase 2 cytoplasmic-like isoform X2 [*Bombyx mori*] |
| DmGS2 | NP_511123.2 | glutamine synthetase 2, isoform C [*Drosophila melanogaster*] |
| BmGDH | NP_001040245.1 | glutamate dehydrogenase [*Bombyx mori*] |
| TcGDH | XP_968936.1 | PREDICTED: glutamate dehydrogenase, mitochondrial [*Tribolium castaneum*] |
| DmGDH | NP_996274.1 | glutamate dehydrogenase, isoform F [*Drosophila melanogaster*] |
| BmGAD1 | XP_004925034.1 | PREDICTED: glutamate decarboxylase-like [*Bombyx mori*] |
| DmGAD1 | NP_523914.2 | glutamic acid decarboxylase 1, isoform A [*Drosophila melanogaster*] |
| BmGAD2 | XP_004932908.1 | PREDICTED: cysteine sulfinic acid decarboxylase-like [*Bombyx mori*] |
| DmGAD2 | NP_001285910.1 | black, isoform C [*Drosophila melanogaster*] |
| BmGABAT | XP_004924846.1 | PREDICTED: 4-aminobutyrate aminotransferase, mitochondrial-like [*Bombyx mori*] |
| TcGABAT | XP_001811587.1 | PREDICTED: 4-aminobutyrate aminotransferase, mitochondrial-like [*Tribolium castaneum*] |
| DmGABAT | NP_649168.2 | CG7433, isoform A [*Drosophila melanogaster*] |
| BmSSADH | XP_004932642.1 | PREDICTED: succinate-semialdehyde dehydrogenase, mitochondrial-like [*Bombyx mori*] |
| TcSSADH | XP_972566.1 | PREDICTED: succinate-semialdehyde dehydrogenase, mitochondrial [*Tribolium castaneum*] |
| DmSSADH | NP_651408.1 | succinic semialdehyde dehydrogenase, isoform A [*Drosophila melanogaster*] |
| BmDAT | NP_001037362.1 | dopamine transporter [*Bombyx mori*] |
| TcDAT | EFA09417.1 | hypothetical protein TcasGA2_TC005219 [*Tribolium castaneum*] |
| DmDAT | AAF76882.1 | dopamine transporter [*Drosophila melanogaster*] |
| BmOAT | XP_004925353.1 | PREDICTED: sodium-dependent noradrenaline transporter [*Bombyx mori*] |
| TcOAT | XP_975356.1 | PREDICTED: sodium-dependent noradrenaline transporter-like isoform X1 [*Tribolium castaneum*] |
| TnOAT | AAL09578.2 | high-affinity octopamine transporter [*Trichoplusia ni*] |
| BmSERT | NP_001037436.1 | serotonin transporter [*Bombyx mori*] |
| TcSERT | XP_968717.1 | PREDICTED: sodium-dependent serotonin transporter [*Tribolium castaneum*] |
| DmSERT | NP_523846.2 | serotonin transporter, isoform A [*Drosophila melanogaster*] |
| CfVMAT | EFN70897.1 | Synaptic vesicular amine transporter [*Camponotus floridanus*] |
| TcVMAT | EEZ98884.1 | hypothetical protein TcasGA2_TC004499 [*Tribolium castaneum*] |
| DmVMAT | NP_001014524.1 | vesicular monoamine transporter, isoform C [*Drosophila melanogaster*] |
| BmChT | XP_004926616.1 | PREDICTED: high-affinity choline transporter 1-like [*Bombyx mori*] |
| TcChT | XP_008201469.1 | PREDICTED: LOW QUALITY PROTEIN: high-affinity choline transporter 1 [*Tribolium castaneum*] |
| DmChT | NP_650743.1 | CG7708, isoform A [*Drosophila melanogaster*] |
| BmVAChT | NP_001275599.1 | vesicular acetylcholine transporter-like [*Bombyx mori*] |
| TcVAChT | XP_975499.1 | PREDICTED: vesicular acetylcholine transporter [*Tribolium castaneum*] |
| DmVAChT | NP_477138.1 | VAChT [*Drosophila melanogaster*] |
| BmEAAT1 | NP_001240824.1 | amino acid transporter-like protein [*Bombyx mori*] |
| DmEAAT1 | NP_477428.1 | excitatory amino acid transporter 1, isoform A [*Drosophila melanogaster*] |
| BmEAAT2 | NP_001240825.1 | glutamate transporter [*Bombyx mori*] |
| DmEAAT2 | NP_001162844.1 | excitatory amino acid transporter 2, isoform C [*Drosophila melanogaster*] |
| BmVGluT | XP_004925576.1 | PREDICTED: vesicular glutamate transporter 2.2-like [*Bombyx mori*] |
| TcVGluT | XP_008192852.1 | PREDICTED: vesicular glutamate transporter 1 [*Tribolium castaneum*] |
| DmVGluT | NP_608681.2 | vesicular glutamate transporter, isoform A [*Drosophila melanogaster*] |
| BmGAT | XP_004923491.1 | PREDICTED: sodium- and chloride-dependent GABA transporter 1-like [*Bombyx mori*] |
| TcGAT | XP_008193348.1 | PREDICTED: sodium- and chloride-dependent GABA transporter 1 [*Tribolium castaneum*] |
| DmGAT | NP_651930.2 | CG1732, isoform A [*Drosophila melanogaster*] |
| BmVGAT | XP_004922590.1 | PREDICTED: vesicular inhibitory amino acid transporter-like [*Bombyx mori*] |
| TcVGAT | XP_008195419.1 | PREDICTED: vesicular inhibitory amino acid transporter [*Tribolium castaneum*] |
| DmVGAT | NP_610938.1 | vesicular GABA transporter [*Drosophila melanogaster*] |
