# Supplementary material for: De novo assembly and characterization of central nervous system transcriptome reveals neurotransmitter signaling systems in the rice striped stem borer, Chilo suppressalis
Source: BMC Genomics. 2015 Jul 15;16(1):525. doi: 10.1186/s12864-015-1742-7 (PMC4501067; doi:10.1186/s12864-015-1742-7)
Supplement: Additional file 12: — Primers used for RT-PCR analysis of enzyme and transporter genes in C. suppressalis . [file 12864_2015_1742_MOESM12_ESM.docx]

**Additional file 12: Primers used for RT-PCR analysis of enzyme and transporter genes in *C. suppressalis*.**

| Primer name | Forward primer (5’-3’) | Reverse primer (5’-3’) |
| --- | --- | --- |
| TH | TACTAGAAGAGGCTCGTGCCCG | GATTCACTCCAGCGAAAGCGGT |
| DDC | TTCGCTAAGGCGATGACTGA | CAGGGTGCTGTTCTTTGACG |
| *ebony* | ACTGTCCAGGGAGACCACCA | CAGCACACCGAAGTCACCAG |
| *tan* | GAAAAGCAAATCGGCAGACG | CAGGGAGATGCCCAGCATAG |
| aaNAT | TGCCAGGCCTCTAGTCAACC | CCAAGCACCTTCAGGATTCG |
| TDC | CGCATGCACCGAATTAGAAA | GAAACGCACTGCCAGCATAC |
| TβH | TTCCGCCAAGGCATAGAACT | TTCCCAATCACCCTGAAACC |
| TPH | ACGTATGCCACGAGTTGCTG | GCACATCCCGAAGAAGATCC |
| TRH | GAGTGGGTCAGGCAAAGGTC | GAGCCCGATCCAAATCTGAG |
| HDC | CGCAGCTATTTGCGACTACG | CTTCGGCTTCCTTGAAATGG |
| ChAT | GGGTACGGACGGGAGTTCAT | CGTATCCGTCGGGAACTACG |
| AChE1 | GAACGAATCGACGACGAAGG | AAGAGGCGACACGGTACTGC |
| AChE2 | ACCATCACGACACCACAACG | GCATGGAAGCAACGATCACA |
| GLS | ACCTGCAAAATCAGGCGTGT | GCTGTGTGGCCAAAGGTTTC |
| GS1 | AATGCCGAGGTCATGTTTGG | CAATCTCCAGCTGGCCTACG |
| GS2 | AGCTGAGGGCCACAACTCTG | TGTAGCGTGCCATCCACAAG |
| GDH | TATTGGGCCCGGTGTAGATG | GGGCGTTCTCTTTGTGGATG |
| GAD1 | TCTGCTGTCGGCAACCTGTA | GCCACCTGGTGCTAGGATTG |
| GAD2 | TAGTAGGCTGGCATAACGGCGAC | TGATGAGGAACGTGGAGCATTGC |
| GABAT | AACAAAAGGCGTGGCTCTGA | AACAAAAGGCGTGGCTCTGA |
| SSDAH | CAGATGAGAGCGTGATTGCCGAC | TGTTCAGCAAGGCTTGCTGCAGC |
| DAT | GCATGACTTGGGCACCATTA | GCTATGGCCGCTGGATAGAC |
| OAT | GGGGCCGTACCATTGTTCTA | CAGGATGGACAGCACCACAT |
| SERT | CGTCCAGAACAAGAGCATCG | AGCAGGAACACGGGACTGAT |
| VMAT | GTTGGCATAGCGATGTTGGA | CATCTCGAGCTGGTGGTGAC |
| ChT | AGAGGTGTTCTGGGCTGCTG | GGCAGGATCATGGAGGTCTG |
| VAChT | CTCGGGTCTCGCCATGATAG | TCAGCCATTGATGCTGAGGA |
| EAAT1 | CGATGAGAAGGACCCGACAC | CATCATCGCCTCCGAAAAAG |
| EAAT2 | GATTCGGTCTTCGCCCCTAC | CTAAGGCTGGCACCGTATCG |
| VGluT | CGTTGGTGCCTATTGGTGGT | TGTGGTCTCATGGTCCAACG |
| GAT | TGATGCCGAACATGTCCAAG | CGATAGCCCGACCAGATACG |
| VGAT | ACACGTACCCGCAGCAAGAT | GTGCCGATCATGAGGTCTCC |
| EF-1 | AAATCGGCGGTATTGGTACG | AAGGGGAGGGAATTCTTGGA |
