# Supplementary material for: De novo assembly and characterization of central nervous system transcriptome reveals neurotransmitter signaling systems in the rice striped stem borer, Chilo suppressalis
Source: BMC Genomics. 2015 Jul 15;16(1):525. doi: 10.1186/s12864-015-1742-7 (PMC4501067; doi:10.1186/s12864-015-1742-7)
Supplement: Additional file 13: — Primers used for qRT-PCR analysis of expression levels of enzyme and transporter genes in C. suppressalis . [file 12864_2015_1742_MOESM13_ESM.docx]

**Additional file 13: Primers used for qRT-PCR analysis of expression levels of enzyme and transporter genes in *C. suppressalis*.**

| Primer name | Forward primer (5’-3’) | Reverse primer (5’-3’) |
| --- | --- | --- |
| TH | CAATTCGATGCTCTCGTGAA | TGGCTCGTATTTGGTCATCA |
| DDC | CTGATTGGACCGACACACAG | AAGGGTATGAGCCCGTTCTT |
| *ebony* | ACTGTCCAGGGAGACCACCA | TTCTCCCGAACAGACCCAGA |
| *tan* | GAAAAGCAAATCGGCAGACG | TGTCGTACGCTCTTCGTCCA |
| aaNAT | GGAAGAATATTCGGCCAGCA | CCAAGCACCTTCAGGATTCG |
| TDC | CGTGAAACTGCGCATCCTAC | GCACCACTGGTCCAATTTCA |
| TβH | TAAATCAGCATGGGCAATGG | CGTTGTAGCGGCGTAAATCA |
| TPH | TCGCCAATACCTTTGAGGAC | ATGAGCAAACCCATTTCCTG |
| TRH | GTGCTCATGTACGGCTCTGA | GGAACACAATTCCCCATGTC |
| HDC | TCGAGAAAGCCGGATTGATT | CTTCCCGAAGGTTGTCGAAC |
| ChAT | GCATCGACAACCATCTGCTG | CGTATCCGTCGGGAACTACG |
| AChE1 | CGACACTGCCTCACTCATGC | CTCCTCCGAACACCCACAAC |
| AChE2 | ACCATCACGACACCACAACG | GCAAACGGTATTCCGGTGAA |
| GLS | ATCCGGCATGGACATGACTC | GCTGTGTGGCCAAAGGTTTC |
| GS1 | TTGGTTTGGGTTGGAACAGG | CATCTTGCGTGAGCCTCAGA |
| GS2 | AGCTGAGGGCCACAACTCTG | AACCACGGCTCCTCATCCTT |
| GDH | CCAAGGCAGGCATCAAAATC | GGCGATCCAGGACATCTCAC |
| GAD1 | TCTGCTGTCGGCAACCTGTA | CATCATCTCCTCCGGCATTC |
| GAD2 | TTTGACCCTCTGGTGCCACT | GCGGTGCGGCTAATAGTTTG |
| GABAT | TGAAGGTGGGGACAACGAAG | GTGAGGGCAGCTCGAAGTGT |
| SSDAH | CGATACGGCTCGTCACATCA | CACCCTGCAGCCATTAAAGC |
| DAT | CCACCTTCTGGGCTCTCATA | TTTAGTGCACGATGCCAGAC |
| OAT | ACCACCCTGGTGAACTGCT | AACAGCATCGCCCAAAGACT |
| SERT | GCCTCACTCTGTGGAAGAGG | GTGCAGAGAGGTGTGTTCCA |
| VMAT | GTGCTCTGGACTCGTCATCA | GCAGTGTCACCGATAGCGTA |
| ChT | GCGAGTGTTGAGCAGCAAGA | GGCAGGATCATGGAGGTCTG |
| VAChT | AGTGTGTGCTGGAGCGTTGA | TCAGCCATTGATGCTGAGGA |
| EAAT1 | CGATGAGAAGGACCCGACAC | CATCATCGCCTCCGAAAAAG |
| EAAT2 | GGCTGCAACATCAACATGGA | CGCTGCTGACGGTACTGATG |
| VGluT | CACAAGATAGGGGCGACCAC | TGTGGTCTCATGGTCCAACG |
| GAT | CGTATCTGGTCGGGCTATCG | GCCGATCATCTCCTTGATGC |
| VGAT | ACACGTACCCGCAGCAAGAT | TTGCATTGGTGACGTTCCAG |
| EF-1 | TGAACCCCCATACAGCGAATCC | TCTCCGTGCCAACCAGAAATAGG |
